# Isolation, Structure Elucidation, and First Total Synthesis of Quinomycins K and L, Two New Octadepsipeptides from the Maowei Sea Mangrove-Derived *Streptomyces* sp. B475

**DOI:** 10.3390/md21030143

**Published:** 2023-02-23

**Authors:** Qinpei Lu, Gang Wu, Xiaomeng Hao, Xinxin Hu, Hao Cai, Xiujun Liu, Xuefu You, Hongwei Guo, Chenghang Sun

**Affiliations:** 1 Guangxi Key Laboratory of Bioactive Molecules Research and Evaluation, Pharmaceutical College, Guangxi Medical University, Nanning 530021, China; 2 Department of Microbial Chemistry, Institute of Medicinal Biotechnology, Chinese Academy of Medical Sciences & Peking Union Medical College, Beijing 100050, China; 3 Beijing Key Laboratory of Antimicrobial Agents, Institute of Medicinal Biotechnology, Chinese Academy of Medical Sciences & Peking Union Medical College, Beijing 100050, China; 4 Department of Pharmacology, Institute of Medicinal Biotechnology, Chinese Academy of Medical Sciences & Peking Union Medical College, Beijing 100050, China; 5 Department of Oncology, Institute of Medicinal Biotechnology, Chinese Academy of Medical Sciences & Peking Union Medical College, Beijing 100050, China; 6 College of Eco-Environmental Engineering, Qinghai University, Xining 810016, China

**Keywords:** Maowei Sea, *Streptomyces* sp., quinomycin, octadepsipeptide, total synthesis

## Abstract

Mangrove actinomycetia have been proven to be one of the promising sources for discovering novel bioactive natural products. Quinomycins K (**1**) and L (**2**), two rare quinomycin-type octadepsipeptides without intra-peptide disulfide or thioacetal bridges, were investigated from the Maowei Sea mangrove-derived *Streptomyces* sp. B475. Their chemical structures, including the absolute configurations of their amino acids, were elucidated by a combination of NMR and tandem MS analysis, electronic circular dichroism (ECD) calculation, advanced Marfey’s method, and further unequivocally confirmed by the first total synthesis. The two compounds displayed no potent antibacterial activity against 37 bacterial pathogens and had no significant cytotoxic activity against H460 lung cancer cells.

## 1. Introduction

As a unique ecosystem with extreme conditions and high biodiversity, mangrove is becoming a rich source for discovering new actinomycetia and novel pharmaceutical compounds. A multitude of bioactive compounds, including the promising compounds salinosporamide A, xiamycins, and indolocarbazoles, have been isolated from mangrove actinomycetia [1,2,3,4]. The Beibu Gulf is located in the northwestern continental shelf area of the South China Sea, from the Leizhou Peninsula, Qiongzhou Strait, and Hainan Island to Vietnam and northward to Guangxi. The mangrove species in this region are very rich, and mangrove in the region is ranked as the second-largest area and accounts for more than 37% of the total mangrove area in China [5]. Recently, our group has discovered many new actinomycetia taxa along with novel bioactive compounds from mangroves in the Beibu Gulf, such as the Maowei Sea [6,7], Beilun Estuary [8,9,10], and Leizhou Peninsula [11].

**Figure 1:**
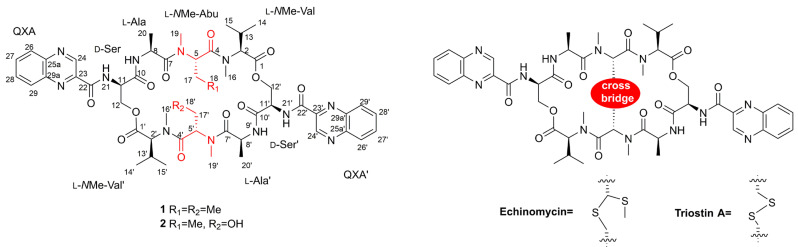
The structures of quinomycins K (**1**) and L (**2**), and known compounds echinomycin and triostin A.

In our previous study, five putative new quinomycin analogues were found by MS/MS-based molecular networking analysis [7]. However, due to a limited amount of sample, only two compounds, quinomycins K (**1**) and L (**2**), were obtained (**1**, 3.2 mg; **2**, 1.8 mg) (Figure 1). Quinomycins are cyclic octadepsipeptides that belong to the quinoxaline family of antibiotics, exhibiting significant antibacterial and antitumor activities due to the bisintercalation of the two quinoxaline rings into DNA [12]. The isolation and synthesis of quinomycins, such as echinomycin and triostin A, have attracted tremendous attention due to their fascinating skeletons and a wide range of biological activities [13,14,15]. In this paper, we present the isolation and complete structural elucidation of quinomycins K (**1**) and L (**2**) based on the combination of spectroscopic analyses, ECD calculations, Marfey’s methods, and the first total synthesis.

## 2. Results and Discussion

Quinomycin K (**1**) was obtained as a white amorphous powder. Its molecular formula was deduced as C_52_H_68_N_12_O_12_ by HR-ESI-MS measurement (*m/z* 1053.5165 [M + H]^+^, calcd for C_52_H_69_N_12_O_12_, 1053.5158), indicating 24 degrees of unsaturation. The IR spectrum suggested the presence of amine or hydroxy (3375 or 3303 cm^−1^), ester carbonyl (1742 cm^−1^), amide carbonyl (1644 cm^−1^), and phenyl (1517 and 1459 cm^−1^) functionalities. The UV maximum absorption wavelength at 242 nm and 317 nm in methanol indicated the presence of a quinoxaline-2-carboxylic acid chromophore [16,17].

**Figure 2:**
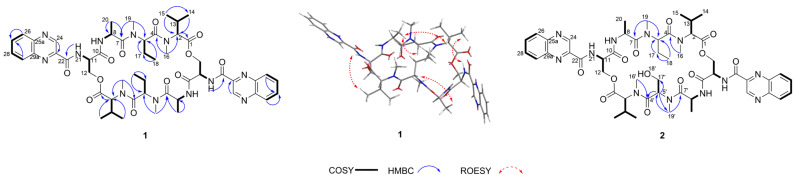
The key COSY, HMBC, and ROESY correlations of **1** and **2**.

**Figure 3:**
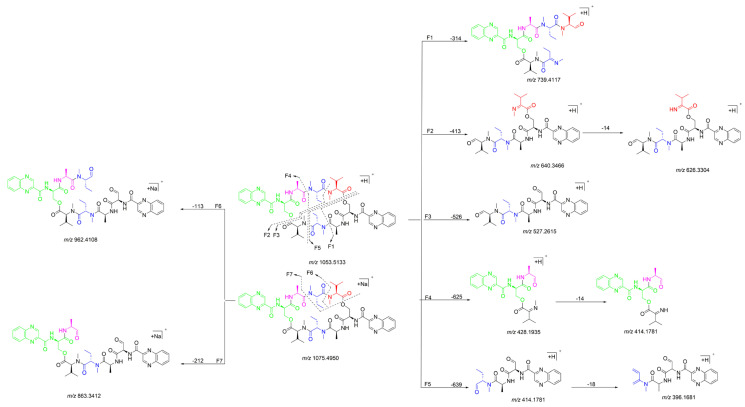
Proposed fragmentation patterns of **1**.

**Figure 4:**
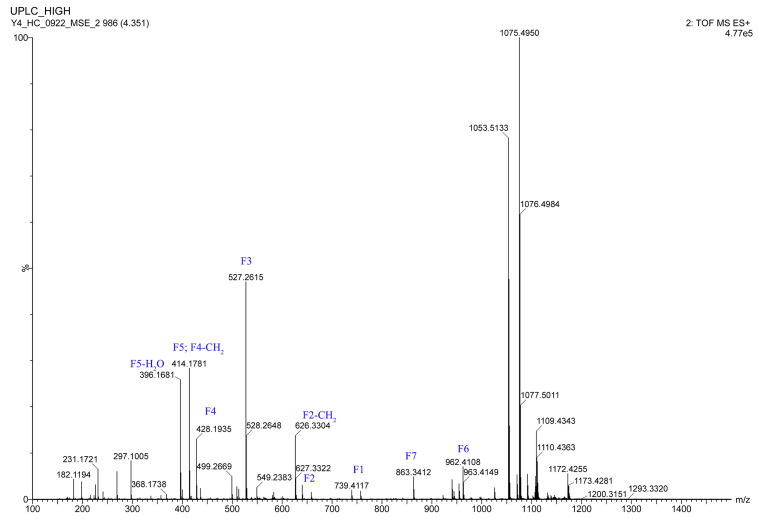
The HRESIMS/MS fragmentation ions of **1**.

The ^1^H NMR data (Table 1) of **1** showed the presence of two exchangeable NH protons (*δ*_H_ 8.70, 8.24), the characteristic signal of quinoxaline-2-carboxylic acid (*δ*_H_ 9.53, 7.97–8.02, 8.19–8.22), two methylamino proton signals (*δ*_H_ 2.92 and 2.85). The SCH_3_ (*δ*_H_≈2.10) [18] and SCH_2_ (*δ*_H_≈3.60) [14] signals were absent in the ^1^H NMR spectrum. The ^13^C NMR spectra, with the aid of 2D NMR, showed the presence of 26 carbons, including four carbonyls (*δ*_C_ 171.4, 170.0, 169.3, 167.9), a quinoxaline-2-carboxylic acid moiety carbon (*δ*_C_ 162.6, 129.0–143.4), one oxygenated methylene (*δ*_C_ 64.4), four aliphatic α-amino carbons (*δ*_C_ 62.1, 54.4, 50.9, 47.2), two methylamino carbons (*δ*_C_ 31.2, 29.5). The number of carbons in the molecular formula has twice as much as those in the ^13^C NMR, indicating that **1** should be a symmetrical dimer with eight amino acid residues. Comparison of NMR spectra of **1** and triostin A [14,19], showed they were similar except for the loss of the Cys residue signal in **1**, suggesting that this Cys residue was replaced by another amino acid group without the intra-peptide disulfide bridge. After examining the NMR data of amino acid residues in **1**, it was inferred that the new amino acid residue was *N*-Me-2-aminobutyric acid (*N*Me-Abu) [20], which was further confirmed by the ^1^H-^1^H COSY correlations of H-5 (*δ*_H_ 5.36)/H_2_-17 (*δ*_H_ 1.66-1.82) and H_2_-17/ H_3_-18 (*δ*_H_ 0.80), and the HMBC correlations from H_3_-18 to C-17 (*δ*_C_ 21.1) and C-5 (*δ*_C_ 54.4), from H_3_-19 (*δ*_H_ 2.85) to C-7 (*δ*_C_ 171.4) and C-5, and from H_3_-16 (*δ*_H_ 2.92) to C-4 (*δ*_C_ 170.0) and C-2 (*δ*_C_ 62.1) (Figure 2). The analysis of MS/MS fragmentation patterns provided additional data to support this assignment (Table 2, Figure 3 and Figure 4). Therefore, the planar structure of **1** was established, as shown in Figure 1.

**Figure 5:**
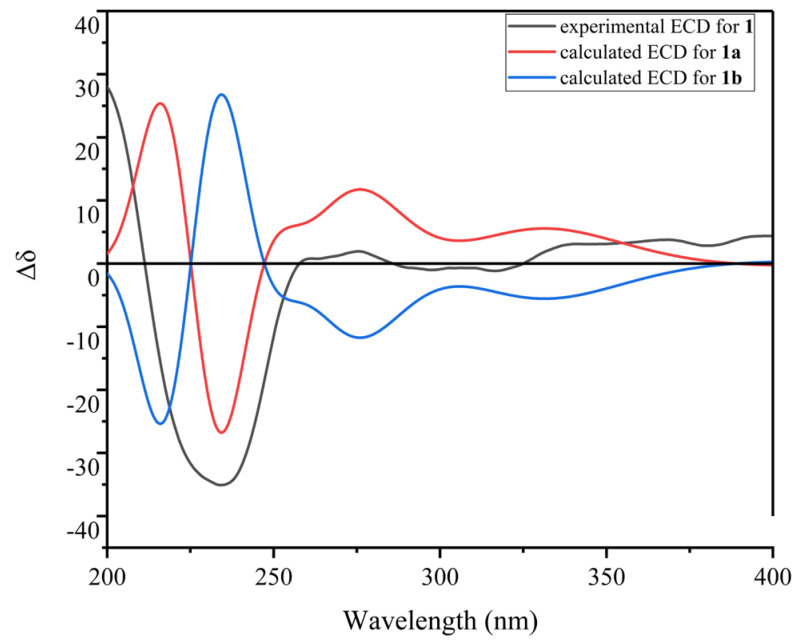
The calculated ECD spectra (**1a**, **1b**) and experimental ECD spectrum of **1**.

**Figure 6:**
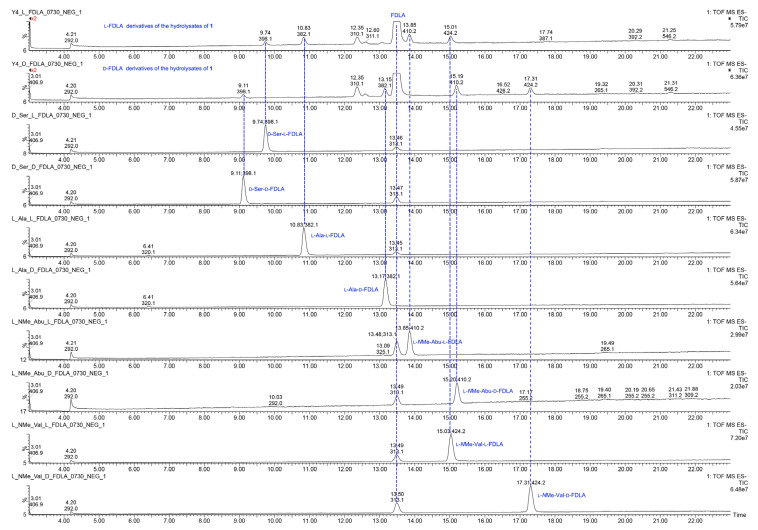
The UPLC-MS chromatograms of l-FDLA and d-FDLA derivatives of the hydrolysates of **1** and amino acid standards.

The relative configuration of **1** was determined by the ROESY spectrum. The key ROESY cross-peak between H_3_-20 (*δ*_H_ 1.29) and H-5, H-13 (*δ*_H_ 2.28-2.35), H_3_-14 (*δ*_H_ 0.95) and H-21′ (*δ*_H_ 8.70) indicated that they were in the same spatial orientation. Thus, two possible isomers (2*S*,5*S*,8*S*,11*R*,2′*S*,5′*S*,8′*S*,11′*R*)-1 (**1a**) and its enantiomer (2*R*,5*R*,8*R*,11*S*,2′*R*,5′*R*,8′*R*,11′*S*)-1 (**1b**) were concluded. To clarify the absolute configurations of **1**, ECD calculation based on the simplified time-dependent density functional theory approach (sTD-DFT) was used, which allowed fast computation of electronic ultraviolet (UV) or circular dichroism (CD) spectra of very large molecules with up to 1000 atoms, such as peptides and proteins [21]. As shown in Figure 5, the calculated ECD spectrum of **1a** was in good agreement with the experimental ECD of **1**, which established the assignment of the absolute configuration of **1** as 2*S*,5*S*,8*S*,11*R*,2′*S*,5′*S*,8′*S*,11′*R*. In addition, the advanced Marfey’s analysis of **1** was performed to determine the absolute configurations of amino acid residues [22]. The presence of d-Ser, l-Ala, l-*N*Me-Abu, and l-*N*Me-Val was unambiguously confirmed by comparison with authentic standards (Table 3, Figure 6 and Figure S11 in Supplementary Materials), which was consistent with the structure of **1a**. 

**Figure 7:**
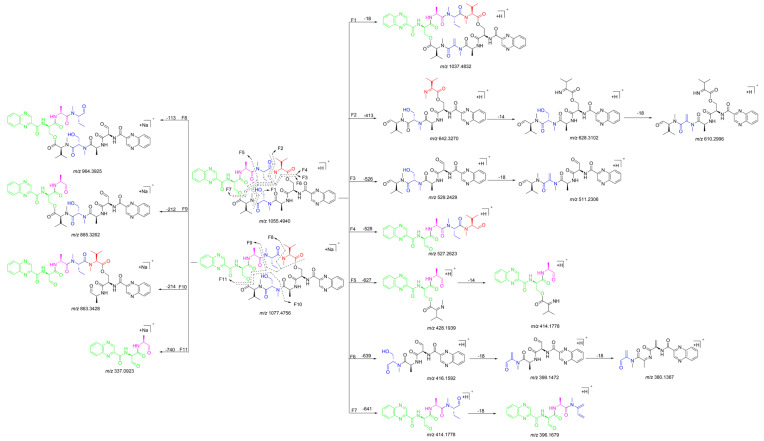
Proposed fragmentation patterns of **2**.

**Figure 8:**
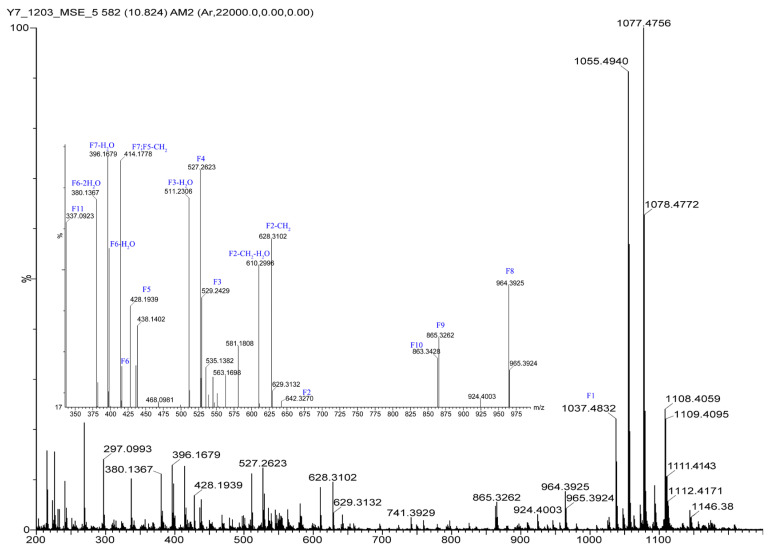
The HRESIMS/MS fragmentation ions of **2**.

**Figure 9:**
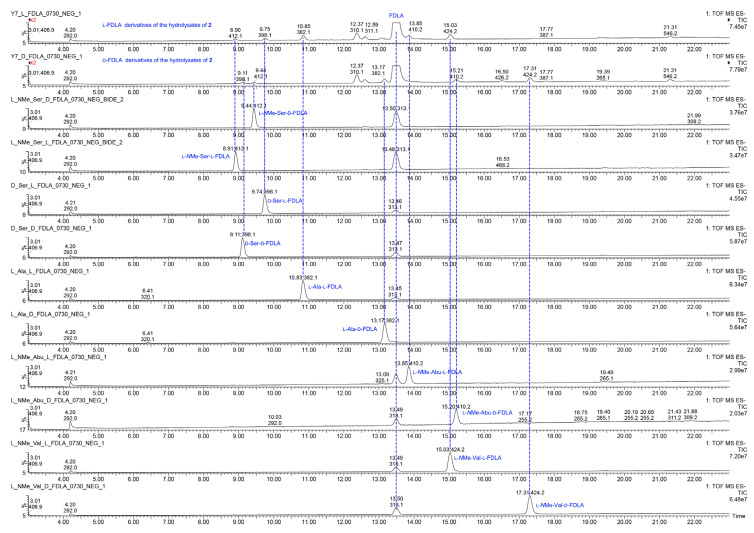
The UPLC-MS chromatograms of l-FDLA and d-FDLA derivatives of the hydrolysates of **2** and amino acid standards.

Quinomycin L (**2**) was obtained as a white amorphous powder. It possessed a molecular formula of C_51_H_66_N_12_O_13_ by the HRESIMS ion at *m/z* 1055.4930 [M + H]^+^ (calcd for C_51_H_67_N_12_O_13_, 1055.4951), two mass units more than compound **1**. According to the characteristics of MS/MS molecular networking [23,24] and our previous study [7], compounds **1** and **2** were neighbor nodes in one cluster, indicating that they had the identical or similar structural fragments in their structure. Therefore, the planar structure of **2** was deduced by analyzing the relationship of MS/MS fragmentation ions between **1** and **2** (Table 2, Figure 3, Figure 7 and Figure 8). Three same fragment ions (863.3412/863.3428, 527.2615/527.2623, and 396.1681/396.1679) existed in **1** and **2**, indicating that the fragment of QXA-(Ser-Ala-*N*Me-Abu-*N*Me-Val-*N*Me-Val′)-QXA′ in **1** was also present in **2**. In addition, the fragment ions at *m/z* 1037.4832 formed by loss of 18 Da neutral mass in the MS/MS spectrum of **2**, indicated the presence of an hydroxyl group in **2**. Lastly, three pairs of ions (Δ = 2 Da) of *m/z* 865.3262/863.3428, 529.2429/527.2623, 398.1472/396.1679 in the ion fragments of **2**, led to speculating that the methyl group (15 Da) in the *N*Me-Abu′ residue of **1** was replaced by a hydroxyl group (17 Da), turning it into an *N*Me-Ser residue in **2**. The NMR data with *δ*_H_ 5.27 (d, *J* = 7.2 Hz), 3.70–3.74 (m), 2.69 (s), and *δ*_C_ 59.0, 59.3 also supported the presence of the *N*Me-Ser residue [25]. In addition, this residue was further confirmed by the cross-peaks of H-5′ (*δ*_H_ 5.27)/H-17′ (*δ*_H_ 3.70–3.74) in the ^1^H-^1^H COSY spectrum, and the HMBC correlations of H-5′ with C-17′ (*δ*_C_ 59.3), C-19′ (*δ*_C_ 29.6), and C-4′ (*δ*_C_ 168.7), H_3_-19′ (*δ*_H_ 2.69) with C-7′ (*δ*_C_ 172.7), C-5′ (*δ*_C_ 59.0), and H_3_-16′ (*δ*_H_ 3.20) with C-4′, C-2′ (*δ*_C_ 61.9). The absolute configuration of compound **2** was determined by amino acid analysis using the advanced Marfey’s method. Comparison of FDLA derivatives of the hydrolysates of **2** with those of appropriate standard amino acids using UPLC-MS techniques indicated l from Ala, *N*Me-Abu, *N*Me-Ser, and *N*Me-Val, and d from Ser in compound **2** (Table 3, Figure 9).

**Scheme 1:**
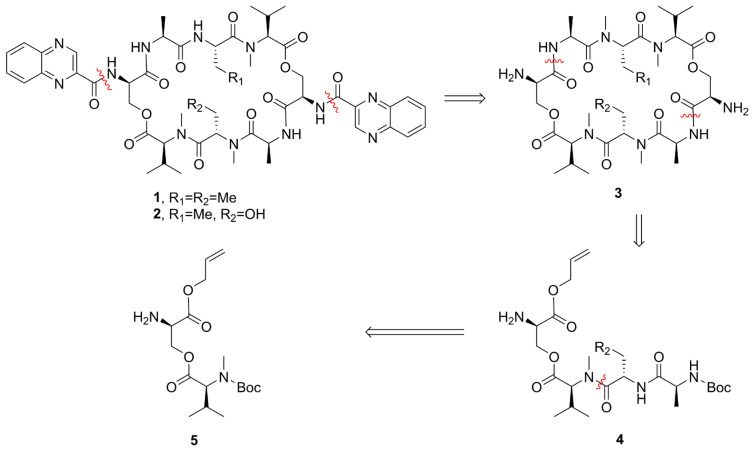
Retrosynthetic analysis of compounds **1** and **2**.

To unequivocally confirm their structure and solve the supply problem for further bioactivity studies, the first total syntheses of compounds **1** and **2** were performed. The solution-phase synthetic procedure described herein will contribute to the development of the synthesis of compounds **1** and **2**. We retrosynthetically disconnected the macrocycle **1** and **2** at the amide bonds linking d-Ser and l-Ala residues (Scheme 1). The intermediate tetrapeptide compound **4** could be accomplished via a sequential peptide coupling approach.

**Scheme 2:**
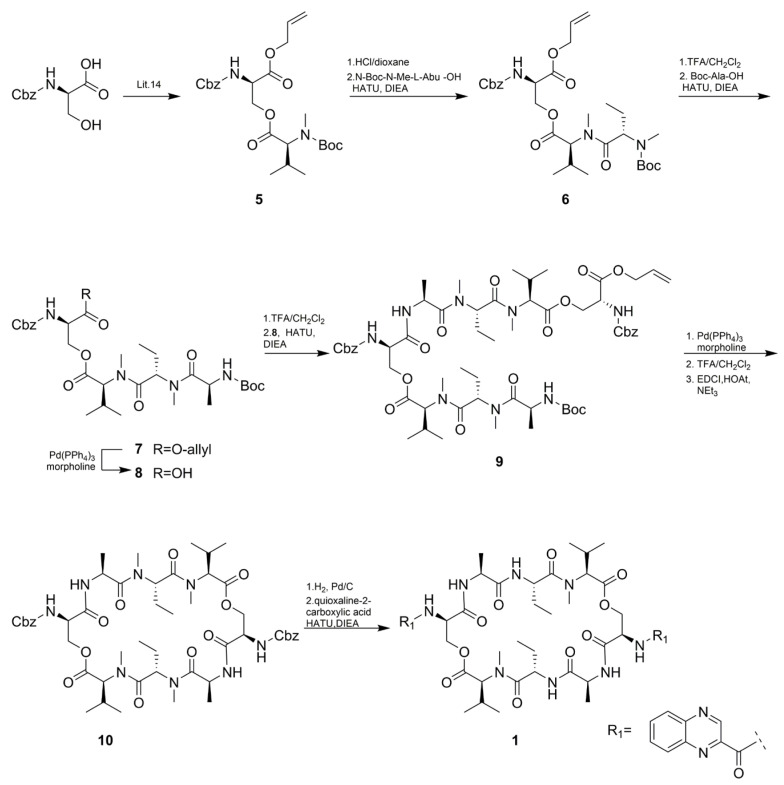
Synthesis of compound **1**.

**Scheme 3:**
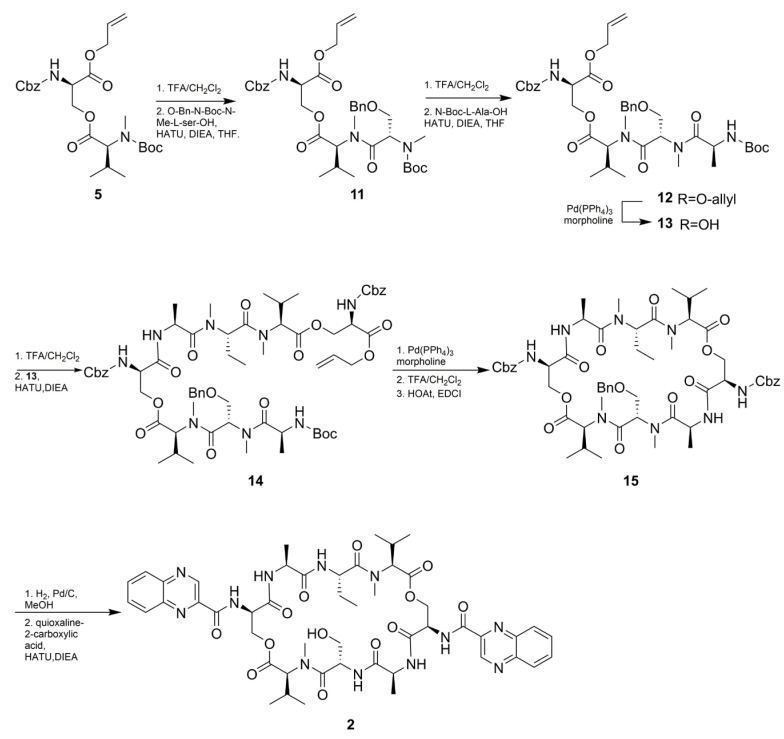
Synthesis of compound **2**.

As shown in Scheme 2, The total synthesis started with the synthesis of Cbz-D-Ser(Boc-MeVal)-OAll (**5**) according to an optimized procedure previously reported by Nagaswa et al. [14,15]. Compound **5** was subsequently condensed with *N*-Boc-methyl-Abu-OH affording the tripeptide **6**. The next step involved the conjugation of *N*-Boc-l-Ala-OH with **6** to give tetradepsipeptide **7** in 60% yield over four steps. Half of the obtained compound **7** was then deallylated to give **8**. Subsequently, *N*-Boc deprotection of **7** was followed by condensation with compound **8** using HATU to afford a linear octadepsipeptide **9**. Compound **9** obtained by deallylation of **8** was subjected to intramolecular amide bond formation to produce cyclic peptide **10**. *N*-Cbz deprotection of **10** was condensed with quinoxaline-2-carboxylic acid to obtain the target compound **1** in 55% yield. The synthetic procedure for compound **2** was similar to compound **1**, except for the O-Bn-*N*-Boc-methyl -Ser-OH instead of *N*-Boc-methyl-Abu-OH (Scheme 3). All the spectroscopic data (^1^H and ^13^C NMR, HRESI-MS, UV, IR, and specific rotation) and UPLC-UV-MS retention times (two different eluting conditions) of the synthetic-**1** and -**2** matched those of the isolated natural products, respectively (Table 1 and Table 4, Figures S31–S38 and Table S1).

Compounds **1**–**2** were evaluated for antibacterial against 37 different bacterial pathogens and cytotoxicity against the H460 lung cancer cells. Compared with the positive control echinomycin, no prominent antibacterial (MIC > 32 μg/mL) and cytotoxic (IC_50_ > 1000 nM) activities were observed in compounds **1**–**2** (Tables S2 and S3)**,** indicating that the cross-linking through a bridge bond may play a key role in the biological effects of quinomycin-type depsipeptides. This result was consistent with the findings from the previous report [15].

## 3. Materials and Methods

### 3.1. General Experimental Procedures

Specific rotations were measured in ACN using a Rudolph Research Analytical Autopol V automatic polarimeter (Rudolph, New Jersey, NJ, USA). ECD spectra were obtained on a JASCO J-815 spectropolarimeter (JASCO, Tokyo, Japan). UV spectra were recorded on a UV-2550 UV/vis spectrophotometer (Shimadzu, Tokyo, Japan). IR spectra were acquired on a Nicolet 5700 FT-IR microscope spectrometer (Thermo Fisher, Madison, WI, USA). NMR spectra were recorded on a Bruker AV III HD 600 NMR instrument (^1^H: 600 MHz, ^13^C: 150 MHz) with TMS as the internal standard (Bruker, Karlsruhe, Germany). HRESIMS and MS/MS data were obtained on a Waters Xevo G2-XS QTof mass spectrometer equipped with ACQUITY UPLC BEH C18 column (2.1 × 100 mm, 1.7 μm) (Waters, Milford, MA, USA). HPLC-MS analysis was performed on a Shimadzu Prominence UFLC-LCMS 2020 system with a YMC-Pack ODS-A column (4.6 × 150 mm, 5 μm). Column chromatography (CC) was carried out on Sephadex LH-20 (Pharmacia, Uppsala, Sweden). MPLC was performed on Biotage Isolera One System (Biotage, Uppsala, Sweden) with a pre-packed column (Yamazen Ultra Pack ODS-SM-50B, 26 × 300 mm, 50 μm). Semi-preparative HPLC was carried out using a Shimadzu LC-20A instrument with an SPD-M20A detector using a semi-preparative YMC-Pack ODS-A column (10 × 250 mm, 5 μm). Unless otherwise noted, reagents and solvents were purchased at the highest commercial quality and used without further purification. In experiments requiring dry solvents, dichloromethane (DCM) and tetrahydrofuran (THF) were purchased from Beijing Yinuokai Technology Co., Ltd. as “Dehydrated”.

### 3.2. Actinomycetia Material

The strain *Streptomyces* sp. B475 was isolated from soil collected from the Maowei Sea Mangrove Reserve, Qinzhou City, Guangxi Zhuang Autonomous Region, China, in July 2017. The 16s rRNA gene sequence data were submitted to GenBank with accession NO. MN199475. Its sequence was similar to that of *Streptomyces seoulensis* NRRL B-24310^T^ (identity: 99.93%). Accordingly, this strain was identified at the genus level as *Streptomyces* sp. The strain was deposited at the Department of Microbial Chemistry, Institute of Medicinal Biotechnology, Chinese Academy of Medical Sciences and Peking Union Medical College.

### 3.3. Fermentation, Extraction, and Isolation

The strain was grown and maintained on an agar plate with ISP2 medium (glucose 4 g/L, yeast extract 4 g/L, malt extract 10 g/L, distilled water 1 L, pH = 7.2) at 28 °C for 7–10 days. The spores of the strain were inoculated into 500 mL Erlenmeyer flasks containing 100 mL of ISP2 medium at 28 °C for three days with shaking at 180 rpm. The 6 L (60 × 100 mL) of fermentation broth was centrifuged at 4300 rpm for 20 min, and the supernatant was extracted three times with ethyl acetate (6 L/time) to give an organic extract. After 20 fermentations, the combined organic extract (8.4 g) was subjected to MPLC column chromatography eluted with MeOH-H_2_O (10:90, 30:70, 50:50, 70:30, 90:10, 100:0, *v/v*) to obtain seven subfractions (Fr.01–Fr.07) based on HPLC-MS analysis. Fr.05 (170 mg) was subjected to Sephadex LH-20 with CH_2_Cl_2_: MeOH (1:1, *v/v*) as the mobile phase to yield five subfractions (Fr.05a–Fr.05e). The fraction Fr.05b was separated by preparative HPLC with a gradient of ACN-H_2_O (55:45, *v/v*, 0–20 min; 55:45 to 60:40, *v/v*, 20–30 min, 2.0 mL/min) to yield compound **1** (3.2 mg) and the mixture of compound **2**. The mixture of **2** was further separated by preparative HPLC using a gradient of MeOH-H_2_O (63:37, *v/v*, 0–30 min; 63:37 to 70:30, *v/v*, 30–45 min, 2.0 mL/min) to obtain compound **2** (1.8 mg).

Quinomycin K (**1**): white amorphous powder; ${\left[\alpha \right]}_{D}^{25}$ −110.0° (c 0.08, ACN); UV (MeOH) λ_max_ (log ε) 242 (4.92), 317 (4.21) nm; ECD (*ϲ* 1.90 × 10^−5^M) λ_max_ (Δε) 234 (−35.08); IR *v*_max_: 3375, 3303, 2968, 2936, 2877, 1742, 1644, 1517, 1492, 1459, 1408, 1180, 1126, 1014 cm^−1^; ^1^H NMR (DMSO-*d_6_*, 600 MHz) and ^13^C NMR (DMSO-*d_6_*, 150 MHz), see Table 1; HRESIMS: *m/z* 1053.5165 [M + H]^+^ (calcd for C_52_H_69_N_12_O_12_, 1053.5158).

Quinomycin L (**2**): white amorphous powder; ${\left[\alpha \right]}_{D}^{25}$ −152.0° (c 0.05, ACN); UV (MeOH) λ_max_ (log ε) 241 (4.59), 323 (3.91) nm; ECD (*ϲ* 1.70 × 10^−5^M) λ_max_ (Δε) 235 (−4.42); IR *v*_max_: 3351, 2918, 2850, 1740, 1637, 1578, 1538, 1492, 1465, 1409, 1260, 1097, 1025 cm^-1^; ^1^H NMR (DMSO-*d_6_*, 600 MHz) and ^13^C NMR (DMSO-*d_6_*, 125 MHz), see Table 4; HRESIMS: *m/z* 1055.4930 [M + H]^+^ (calcd for C_51_H_67_N_12_O_13_, 1055.4951).

### 3.4. ECD Calculation of Compound **1**

Conformational search and geometry optimizations for **1a** were performed on Molclus 1.9.9 program [26] by invoking xtb 6.3.3 program [27], Gaussian 16 packages [28], and ORCA 4.2.1 program [29] as described previously [30,31,32]. Firstly, the original structure was used to perform molecular dynamics (MD) simulations in the xtb 6.3.3 program with GFN0-xTB [33], the thermostat temperature was set at 400 K, and the total run time of simulation was 150 ps. These conformers were subjected to semi-empirical geometry optimization using the GFN0-xTB and GFN2-xTB [34] with the GBSA model in the MeOH method successively, the xTB geometries with a difference of distance geometries and energies within 0.5 were clustered by the isostat module in the Molclus program. Then, the clustered geometries within an energy window of 3 kcal/mol were subjected to a DFT geometry optimization and frequency analyses at B3LYP-D3(BJ)/6-31G level of theory with DFT-D3 dispersion correction [35] using Gaussian 16 program, and subsequently subject to ORCA program for calculating high precision single point energy at RI-PWPB95-D3(BJ)/def2-TZVPP level with SMD solvent model in MeOH. Conformer’s clustering, their relative Gibbs Free Energy, and Boltzmann distribution in room temperature (298.15 K) were obtained from the isostat module in the Molclus program. Those conformers with Boltzmann distribution over 2% population were subjected to subsequent calculations.

ECD calculation was performed using the simplified time-dependent density functional theory (sTD-DFT) approach [21] at the wB97X-D3/def2-SV(P) level of theory in MeOH with the SMD model in the ORCA program. The calculated ECD curves of each conformer and their Boltzmann-weighed ECD curves were generated using Multiwfn 3.7 software [36]. Finally, the ECD of its enantiomer **1b** was generated and plotted together with the experimental ECD of **1**, calculated ECD of **1a** using Origin 2018 software (OriginLab, Northampton, MA, USA).

### 3.5. Advanced Marfey’s Analysis of Compounds **1-2**

Analysis was carried out following the published method [22]. Samples of **1**–**2** (each 0.12 mg) were dissolved in 6M HCl (0.2 mL) and heated to 110 °C in a sealed vial for 16 h. The hydrolysates were concentrated under dry N_2_ at 40 °C, dissolved in 0.1 mL H_2_O, and divided into two portions (each 50 μL). Each portion was treated with 20 μL of 1M NaHCO_3_, and then we added 100 μL of 1% l-FDLA and 1% d-FDLA in acetone, respectively. The mixtures were vortexed and incubated at 37 °C for 1 h. After cooling to room temperature, the reaction mixture was quenched by adding 20 μL of 1M HCl and diluted with 200 μL of CH_3_CN. A total of 20 μL of the resulting solutions were diluted again with 800 μL of CH_3_CN and then centrifuged at 12,000 rpm for 10 min. The supernatants (1 μL) were analyzed by UPLC-HRESI-MS using an ACQUITY UPLC BEH C18 column (2.1 × 100 mm, 1.7 μm, 0.3 mL/min) with gradient elution (solvent A: water with 0.1% acetic acid; solvent B: CH_3_CN containing 0.1% acetic acid; 5% B, 0–3 min, 20–60% B, 3–23 min, UV detection: 340 nm; column temperature: 40 °C). The MS scanned *m/z* range 250–1100 in negative mode. Commercial standards of d-Ser, l-Ala, l-*N*Me-Abu, l-*N*Me-Ser, and l-*N*Me-Val were derivatized and analyzed in the same way. The absolute configurations of amino acids were established by comparison of the retention times of the l- and d-FDLA derivatives of corresponding amino acids.

### 3.6. The Synthesis of **1** and **2**

#### 3.6.1. *N*-Cbz-d-Ser[*N*-Boc-*N*-Me-l-Abu -*N*-Me-l-Val]-OAll (**6**)

The *N*-Cbz-d-Ser(*N*-Boc-*N*-Me-l-Val)-OAll **5** (1.18 g, 2.4 mmol, 1.1 equiv) was dissolved in 4 M HCl/dioxane (10 mL, 60 mmol) at 0 °C. Then the resulting solution was warmed to room temperature and stirred at that temperature for 2 h. The mixture was diluted with EtOAc (200 mL) and sat. NaHCO_3_ aq. (100 mL). The two layers were separated, and the organic layer was washed with brine (100 mL) and dried over Na_2_SO_4_, and the solvent was removed under reduced pressure to obtain the residue as a white solid. ^1^H NMR (500 MHz, CDCl_3_) *δ*_H_ 9.87 (s, 1H), 9.64 (s, 1H), 7.37–7.26 (m, 5H), 6.88 (d, *J* = 8.2 Hz, 1H), 5.87 (ddt, *J* = 16.5, 11.0, 5.7 Hz, 1H), 5.31 (d, *J* = 17.2 Hz, 1H), 5.22 (d, *J* = 10.4 Hz, 1H), 5.10 (s, 2H), 4.74 (dd, *J* = 11.2, 4.1 Hz, 1H), 4.69 (dt, *J* = 7.5, 3.2 Hz, 1H), 4.62 (d, *J* = 5.7 Hz, 2H), 4.36 (dd, *J* = 11.1, 2.9 Hz, 1H), 3.52 (s, 1H), 2.65 (s, 3H), 2.57–2.44 (m, 1H), 1.07 (d, *J* = 6.8 Hz, 4H), 1.04 (d, *J* = 6.7 Hz, 3H). ^13^C NMR (125 MHz, CDCl_3_) *δ*_C_ 168.9, 165.9, 156.4, 136.4, 131.3, 128.5, 128.1, 128.0, 119.3, 67.4, 67.0, 66.7, 65.6, 53.0, 33.0, 29.7, 19.6, 17.8.

Then, to a solution of the residue (4 g, 10.19 mmol), *N*-Boc-*N*-Me-l-Abu -OH (2.21 g, 10.19 mmol, 1 equiv), and DIEA (1.86 g, 11.21 mmol) in DMF (25 mL) was added HATU (0.91 g, 3.3 mmol, 1.5 equiv) at 0 °C and stirred at room temperature overnight. The mixture was diluted with EtOAc (150 mL) and water (150 mL). The two layers were separated, and the organic layer was washed with HCl (1 mol/L, 100 mL) and brine (100 mL) and dried over Na_2_SO_4_. The solvent was removed under reduced pressure, and the residue was purified by flash column chromatography on silica gel eluted with *n*-hexane: EtOAc (80:20, *v*/*v*) to afford the target compound **6** as a colorless oil (5 g, 82.9% yield). ^1^H NMR (500 MHz, CDCl_3_) *δ*_H_ 7.06 (q, *J* = 12.8, 9.0 Hz, 5H), 6.17-5.99 (m, 1H), 5.62 (ddt, *J* = 18.7, 12.5, 6.8 Hz, 1H), 5.10–4.78 (m, 4H), 4.72–4.48 (m, 1H), 4.46–4.13 (m, 4H), 2.80–2.69 (m, 2H), 2.63 (dd, *J* = 16.5, 11.7 Hz, 1H), 2.59-2.29 (m, 7H), 2.04–1.83 (m, 1H), 1.50 (dddt, *J* = 30.8, 22.6, 15.1, 7.9 Hz, 2H), 1.26–1.07 (m, 9H), 0.79–0.67 (m, 3H), 0.66–0.49 (m, 7H).

#### 3.6.2. N-Cbz-d-Ser[N-Boc-l-Ala-N-Boc-N-Me-l-Abu-N-Me-l-Val]-OAll (**7**)

To a solution of *N*-Cbz-d-Ser[*N*-Boc-*N*-Me-l-Abu-*N*-Me-l-Val]-OAll **6** (4 g, 6.76 mmol) in DCM (9 mL) was added TFA (3 mL) at 0 °C. Then the resulting solution was warmed to room temperature and stirred for 1 h. The solvent was removed under reduced pressure, then the residue was diluted with EtOAc (200 mL) and sat. NaHCO_3_ aq. (100 mL). The two layers were separated, and the organic layer was washed with brine (100 mL) and dried over Na_2_SO_4_; the solvent was removed under reduced pressure to get the residue as a white solid. ^1^H NMR (500 MHz, CDCl_3_) *δ*_H_ 6.65–6.54 (m, 5H), 6.31 (d, *J* = 7.7 Hz, 1H), 5.58 (d, *J* = 7.5 Hz, 1H), 4.84-4.76 (m, 1H), 4.59 (d, *J* = 8.2 Hz, 1H), 4.36 (q, *J* = 7.8, 5.2 Hz, 2H), 4.01–3.92 (m, 1H), 3.88–3.74 (m, 2H), 3.71–3.59 (m, 1H), 1.49 (dtt, *J* = 19.1, 12.6, 5.9 Hz, 1H), 0.94 (d, *J* = 6.6 Hz, 1H), 0.74–0.61 (m, 9H), 0.50 (dt, *J* = 22.6, 5.7 Hz, 3H), 0.30–0.02 (m, 8H). ^13^C NMR (125 MHz, CDCl_3_) *δ*_C_ 172.2, 169.9, 169.6, 168.8, 161.3, 154.8, 154.0, 135.5, 127.0, 126.6, 126.5, 64.9, 62.7, 60.9, 53.1, 52.9, 52.0, 45.4, 34.8, 29.9, 29.6, 28.4, 26.8, 25.8, 20.2, 18.4, 17.5, 16.3, 8.4.

Then, to a solution of the residue (2.5 g, 5.09 mmol), *N*-Boc-*N*-l-Ala-OH (1.06 g, 5.09 mmol, 1 equiv), and DIEA (1.01mL, 6.01 mmol) in DMF (15 mL) was added HATU (2.12 g, 5.59 mmol) at 0 °C and stirred at room temperature overnight. The mixture was diluted with EtOAc (150 mL) and water (150 mL). The two layers were separated, and the organic layer was washed with HCl (1 mol/L, 100 mL) and brine (100 mL) and dried over Na_2_SO_4_. The solvent was removed under reduced pressure, and the residue was purified by flash column chromatography on silica gel eluted with *n*-hexane: EtOAc (50:50, *v/v*) to afford the target compound **7** as a white solid (2.8 g, 83.1% yield).

#### 3.6.3. N-Cbz-d-Ser[N-Boc-l-Ala-N-Boc-N-Me-l-Abu-N-Me-l-Val]-OH (**8**)

To a solution of *N*-Cbz-d-Ser[*N*-Boc-l-Ala-*N*-Boc-*N*-Me-l-Abu-*N*-Me-l-Val]-OAll **7** (1 g, 1.51 mmol), Pd(PPh_3_)_4_ (3.5 mg) in DCM (10 mL) was added morpholine (0.46 ml, 5.28 mmol) and stirred at room temperature for 1 h. Then the mixture was diluted with EtOAc (100 mL) and sat. NH_4_Cl aq. (100 mL). The two layers were separated, and the water layer was extracted with AcOEt (100 mL). The combined organic layers were washed with brine (100 mL) and dried over Na_2_SO_4_. The solvent was removed under reduced pressure, and the residue was purified by flash column chromatography on silica gel eluted with DCM: MeOH (100:1, *v*/*v*) to afford the target compound **8** as a white solid (800 mg, 85.2% yield). 

#### 3.6.4. N-Cbz-d-Ser[N-Cbz-d-Ser(N-Boc-l-Ala-N-Boc-N-Me-l-Abu-N-Me-l-Val)-l-Ala-N-Boc-N-Me-l-Abu-N-Me-l-Val]-OAll (**9**)

The *N*-Cbz-d-Ser[*N*-Boc-l-Ala-*N*-Boc-*N*-Me-l-Abu-*N*-Me-l-Val]-OAll **7** (1 g, 1.51 mmol) in TFA:DCM (1:3, 12 mL) at 0 °C. Then the resulting solution was warmed to room temperature and stirred for 1 h. The solvent was removed under reduced pressure. Then to a solution of the residue (737 mg, 1.13 mmol), *N*-Cbz-d-Ser[*N*-Boc-l-Ala-*N*-Boc-*N*-Me-l-Abu-*N*-Me-l-Val]-OH **8** (700 mg, 1.13 mmol), and DIEA (0.17 mL, 1.13 mmol) in DMF (10 mL) was added HATU (427 mg, 1.13 mmol) at 0 °C and stirred at room temperature overnight. The mixture was diluted with EtOAc (150 mL) and water (150 mL). The two layers were separated, and the organic layer was washed with HCl (1 mol/L, 100 mL) and brine (100 mL) and dried over Na_2_SO_4_. The solvent was removed under reduced pressure. The residue was purified by flash column chromatography on silica gel with DCM: MeOH (100:1, *v/v*) as the mobile phase to afford the target compound **9** as a white solid (1 g, 70.4% yield).

#### 3.6.5. Cbz-Cyclic Peptide (**10**)

To a solution of *N*-Cbz-d-Ser[*N*-Cbz-d-Ser(*N*-Boc-l-Ala-*N*-Boc-*N*-Me-l-Abu-*N*-Me-l-Val)- l-Ala-*N*-Boc-*N*-Me-l-Abu-*N*-Me-l-Val]-OAll **9** (1 g, 1.51 mmol), Pd(PPh_3_)_4_ (3.5 mg) in DCM (10 mL) was added morpholine (0.46 mL, 5.28 mmol) and stirred at room temperature for 1 h. Then the mixture was diluted with EtOAc (100 mL) and sat. NH_4_Cl aq. (100 mL). The two layers were separated, and the water layer was extracted with EtOAc (100 mL). The combined organic layers were washed with brine (100 mL) and dried over Na_2_SO_4_; the solvent was removed under reduced pressure. The residue was dissolved in TFA: DCM (1:3, 12 mL) at room temperature and stirred for 1 h. The solvent was removed under reduced pressure. The residue was dissolved in DMF: DCM (1:9, 100 mL) and adjusted to pH 7 with DIEA. The HOAt (60.8 mg, 0.446 mmol) and EDCI (85.7 mg, 0.446 mmol) were added to the mixture at 0 °C and then warmed to room temperature and stirred for 24 h. The mixture was washed with HCl (1 mol/L, 100 mL), sat. NaHCO_3_ (100 mL) and brine (100 mL) and dried over Na_2_SO_4_. The solvent was removed under reduced pressure, and the residue was purified by flash column chromatography on silica gel using DCM: MeOH (100:1, *v/v*) as the mobile phase to afford the target compound **10** as a colorless oil (200 mg, 40.6% yield).

#### 3.6.6. Cyclic Peptide (**1**)

A solution of Cbz-cyclic peptide **10** (50 mg, 0.05 mmol) and 20% Pd(OH)_2_/C (5 mg, 10 wt %) in MeOH (5 mL) was stirred at room temperature under an H_2_ atmosphere (1 atm) for 12 h. Then, the mixture was filtered, and the filtrate was concentrated under reduced pressure. To a solution of 2- quinoxalinecarboxylic acid (17 mg, 0.1 mmol, 2 equiv) in DMF (2 mL) was added the residue dissolved in DMF (1 mL). After cooling to 0 °C, The HATU (36 mg, 0.1 mmol, 2 equiv) and DIEA (16 μL, 0.1 mmol, 2 equiv) were added to the mixture, and the solution was stirred at room temperature for 2 h. The mixture was diluted with CH_2_Cl_2_ (50 mL) and washed with water (50 mL). The organic layer was separated, washed with brine (50 mL), dried over Na_2_SO_4_, and concentrated under reduced pressure. The residue was purified by pre-HPLC eluted with ACN-H_2_O (50:50 to 60:40, *v*/*v*, 0–30 min, 2.0 mL/min) to afford the target compound **1** (10 mg, 20% yield) as a white solid. ^1^H NMR (DMSO-*d_6_*, 600 MHz) and ^13^C NMR (DMSO-*d_6_*, 150 MHz), see Table 1; HRESIMS: *m/z* 1053.5137 [M + H]^+^ (calcd for C_52_H_69_N_12_O_12_, 1053.5158). ${\left[\alpha \right]}_{D}^{25}$ −110.0° (c 0.08, ACN); UV (MeOH) λ_max_ (log ε) 242 (4.82), 324 (4.10) nm; IR *v*_max_: 3378, 3312, 2968, 2937, 2877, 1743, 1644, 1517, 1492, 1465, 1408, 1180, 1128, 1014 cm^−1^. 

#### 3.6.7. N-Cbz-d-Ser(N-Boc-N-Me-O-Bn-l-Ser-N-Me-l-Val)-OAll (**11**)

To a solution of the *N*-Cbz-d-Ser(*N*-Boc-*N*-Me-l-Val)-OAll **5** (3 g, 7.64 mmol), *N*-Boc-*N*-Me-O-Bn-l-Abu-OH (2.36 g, 7.64 mmol, 1 equiv), and DIEA (1.4 mL, 8.41 mmol) in DMF (25 mL) was added HATU (3.19 g, 8.41 mmol) at 0 °C and stirred at room temperature overnight. The mixture was diluted with EtOAc (150 mL), and the organic layer was washed with HCl (1 mol/L, 100 mL) and brine (100 mL) and dried over Na_2_SO_4_. The solvent was removed under reduced pressure, and the residue was purified by flash column chromatography on silica gel eluted with *n*-hexane: EtOAc (70:30, *v/v*) to afford the target compound **11** as a white solid (3.5 g, 66.9% yield).

#### 3.6.8. N-Cbz-d-Ser[N-Boc-N-l-Ala-N-Boc-N-Me-O-Bn-l-Ser-N-Me-l-Val]-OAll (**12**)

To a solution of *N*-Cbz-d-Ser[*N*-Boc-*N*-Me-O-Bn-l-Ser-*N*-Me-l-Val]-OAll **11** (2.6 g, 4.52 mmol) in DCM (9 mL) was added TFA (3 mL) at 0 °C. Then the resulting solution was warmed to room temperature and stirred for 1 h. The solvent was removed under reduced pressure, and then the residue was diluted with EtOAc (200 mL) and sat. NaHCO_3_ aq. (100 mL). The two layers were separated, and the organic layer was washed with brine (100 mL) and dried over Na_2_SO_4_, and the solvent was removed under reduced pressure to get the residue as a white solid. Then to a solution of the residue (2.2 g), *N*-Boc-*N*-l-Ala-OH (942 mg, 4.98 mmol, 1 equiv), and DIEA (0.82 mL, 4.98 mmol) in DMF (15 mL) was added HATU (1.89 g, 4.98 mmol) at 0 °C and stirred at room temperature overnight. The mixture was diluted with EtOAc (150 mL), and the organic layer was washed with HCl (1 mol/L, 100 mL) and brine (100 mL) and dried over Na_2_SO_4_. The solvent was removed under reduced pressure, and the residue was purified by flash column chromatography on silica gel eluted with *n*-hexane: EtOAc (50:50, *v/v*) to afford the target compound **12** as a colorless oil (2.5 g, 72.0% yield).

#### 3.6.9. N-Cbz-d-Ser(N-Boc-l-Ala-N-Boc-N-Me-O-Bn-l-Ser-N-Me-l-Val)-OH (**13**)

To a solution of *N*-Cbz-d-Ser(*N*-Boc-l-Ala-*N*-Boc-*N*-Me-O-Bn-l-Ser-*N*-Me-l-Val)-OAll **12** (1.5 g, 1.51 mmol), Pd(PPh_3_)_4_ (26 mg) in DCM (10 mL) was added morpholine (0.21 mL, 2.31 mmol) and stirred at room temperature for 1 h. Then the mixture was diluted with EtOAc (100 mL) and sat. NH_4_Cl aq. (100 mL). The two layers were separated, and the water layer was extracted with EtOAc (100 mL). The combined organic layers were washed with brine (100 mL) and dried over Na_2_SO_4_. The solvent was removed under reduced pressure, and the residue was purified by flash column chromatography on silica gel eluted with DCM: MeOH (100:1, *v*/*v*) to afford the target compound **13** (1.3 g, 91.5% yield).

#### 3.6.10. N-Cbz-d-Ser[N-Cbz-d-Ser(N-Boc-l-Ala-N-Boc-N-Me-l-Abu-N-Me-l-Val)- l-Ala-N-Boc-N-Me-l-Abu-N-Me-l-Val]-OAll (**14**)

Target compound **14** (1.1 g, 0.44 mmol, 46.8% yield over two steps) was synthesized according to the procedure for the synthesis of compound **1** by using *N*-Cbz-d-Ser(*N*-Boc-l-Ala-*N*-Boc-*N*-Me-O-Bn-l-Ser-*N*-Me-l-Val)-OH **13** (1.2 g, 1.93 mmol), *N*-Cbz-d-Ser(*N*-Me-l-Abu-*N*-Me-l-Val)-OAll **12** (1.38 g, 1.93 mmol, 1 equiv), DIEA(0.32 mL, 1.93 mmol, 1 equiv), DMF (10 mL) in step one and morpholine (0.19 ml, 1.93 mmol, 1 equiv), Pd(PPh_3_)_4_ (22.3 mg, 1%), CH_2_Cl_2_ (20 mL) in step two.

#### 3.6.11. Cbz-Cyclic Peptide (**15**)

Target compound **15** (270.6 mg, 0.25 mmol, 30% yield over two steps) was synthesized according to the procedure for the synthesis of compound **1** by using *N*-Boc-octadepsipeptide-OAll **14** (1 g, 0.82 mmol), TFA (2 mL) and CH_2_Cl_2_ (6 mL) in step one and HOAt (111.5 mg, 0.82 mmol, 1 equiv), CH_2_Cl_2_ (350 mL), DIPEA (0.14 mL, 0.82 mmol, 1 equiv) and EDCI·HCl (157.1 mg, 0.82 mmol, 1 equiv) in step two.

#### 3.6.12. Cyclic Peptide (**2**)

Target compound **2** (white solid, 30 mg, 0.03 mmol, 15.6% yield over two steps) was synthesized according to the procedure for the synthesis of compound **1** by using Cbz-bicyclic peptide **15** (200 mg, 0.18 mmol), Pd/C (20 mg, 10%) and methanol (10 mL) in step one and quinoxaline-2-carboxylic acid (126.6 mg, 0.73 mmol, 4 equiv), DMF (5 mL), DIPEA (0.13 mL, 0.73 mmol, 4 equiv) and HATU (275.7 mg, 0.73 mmol, 4 equiv) in step two. ^1^H NMR (DMSO-*d_6_*, 600 MHz) and ^13^C NMR (DMSO-*d_6_*, 150 MHz), see Table 4; HRESIMS: *m/z* 1055.4944 [M + H]^+^ (calcd for C_51_H_67_N_12_O_13_, 1055.4951). ${\left[\alpha \right]}_{D}^{25}$ −155.0° (c 0.05, ACN); UV (MeOH) λ_max_ (log ε) 242 (4.54), 317 (3.83) nm; IR *v*_max_: 3304, 2938, 2872, 1741, 1640, 1536, 1492, 1462, 1412, 1261, 1109, 1052 cm^−1^.

### 3.7. Biological Activity Test

#### 3.7.1. Antibacterial Assay

The MIC values of the synthetic-**1** and synthetic-**2** were determined by using the agar dilution assay at various concentrations of 64, 32, 16, 8, 4, 2, 1, 0.5, 0.25, 0.12, 0.06, and 0.03 μg/mL described by the Clinical Laboratory Standards Institute (CLSI) [37]. Bacterial strains were purchased from the ATCC collection or isolated from the hospitals in China, including *Staphylococcus epidermidis* (ATCC 12228, 18-1, and 17-13), *Staphylococcus aureus* (ATCC 29213, ATCC 33591, ATCC 43300, ATCC 700698, 15, 18-2, and 18-3), *Enterococcus faecalis* (ATCC 29212, ATCC 51299, ATCC 51575, and 18-6), *Enterococcus faecium* (ATCC 700221, 18-4, and 15-6), *Escherichia coli* (ATCC 25922, ATCC 35218, 1515, 18-1, and 18-4), *Klebsiella pneumoniae* (ATCC 700603, ATCC BAA-2146, 7, 18-2, and 18-8), *Pseudomonas aeruginosa* (ATCC 27853 and PAO1), *Acinetobacter baumannii* (ATCC 19606 and 16-33), *Enterobacter cloacae* ATCC 43560, *Enterobacter aerogenes* ATCC 13048*, Serratia marcescens* ATCC 21074, *Proteus mirabilis* ATCC 49565, *Stenotrophomonas maltophilia* ATCC 13636, *Shigella flexneri* ATCC 12022. Levofloxacin and echinomycin were used as the positive controls. The medium of the agar dilution method was Mueller–Hinton agar. Suspensions of each microorganism were prepared to contain approximately 10^6^ colony forming units (CFU)/mL and applied to plates with serially diluted compounds to be tested by multipoint inoculator and then incubated at 37 °C overnight. The MIC was considered as the lowest concentration that completely inhibited the growth on agar plates.

#### 3.7.2. Cytotoxicity Assay 

H460 lung cancer cells were obtained from the Department of Oncology, Institute of Medicinal Biotechnology, Chinese Academy of Medical Sciences and Peking Union Medical College. The 4.0 × 10^3^ H460 lung cancer cells suspended in 100 μL 1640 medium were plated in 96-well plates. After 24 h of growth, cells were treated with different concentrations of echinomycin, synthetic-**1,** and synthetic-**2** for 72 h. Then, cells were incubated with MTT solution (10 μL, 5 mg/mL) for an additional 4 h. After removing the solution, 150 μL of dimethyl sulfoxide (DMSO) was added to each well for 10 min. The absorbance (A) was detected at 570 nm. Untreated cells served as control. Statistical analysis was performed using SPSS software version 26.0 (IBM, Amonk, NY, USA).

## 4. Conclusions

In conclusion, chemical investigation of the Beibu Gulf mangrove-derived *Streptomyces* sp. B475 led to the isolation of two novel quinomycin-type octadepsipeptides, quinomycins K (**1**) and L (**2**), which were characterized by the loss of intra-peptide disulfide or thioacetal bridge. Their planar structures and absolute configurations were elucidated by detailed NMR, MS spectroscopic analyses, calculated ECD analyses, advanced Marfey’s method, and total syntheses. Compounds **1** and **2** did not show potent antibacterial and antitumor activities but displayed the structure–activity relationship of quinomycins. The presence of cross-linking through a bridge bond should contribute to the potent biological activity of quinomycin-type octadepsipeptides.

## Figures and Tables

**Table 1:** The ^1^H (600 MHz, DMSO-*d_6_*) and ^13^C (150 MHz, DMSO-*d_6_*) NMR data of natural- and synthetic-**1**.

Position	Natural-1		Synthetic-1	
	*δ*_H_ (multi, *J*, Hz)	*δ*_C_	*δ*_H_ (multi, *J*, Hz)	*δ*_C_
QXA/QXA′				
23/23′		143.1		143.1
24/24′	9.53 (s)	143.4	9.53 (s)	143.4
26/26′	8.19–8.22 (m)	129.0	8.18–8.21 (m)	129.0
27/27′	7.97–8.02 (m)	132.0	7.96–8.02 (m)	132.1
28/28′	7.97–8.02 (m)	131.6	7.96–8.02 (m)	131.6
29/29′	8.19–8.22 (m)	129.2	8.18–8.22 (m)	129.2
25a/25a′		143.1		143.1
29a/29a′		139.4		139.4
22/22′		162.6		162.5
Ser/Ser′				
21-NH/21′-NH	8.70 (d, 8.4)		8.69 (d, 8.4)	
11/11′	4.98 (m)	50.9	4.99 (m)	50.9
12/12′	4.43 (m)	64.4 ^a^	4.43 (m)	64.4
10/10′		167.9		167.9
Ala/Ala′				
9-NH/9′-NH	8.24 (m)		8.23 (m)	
8/8′	4.61 (m)	47.2 ^a^	4.62 (m)	47.4 ^a^
20/20′	1.29 (d, 7.2)	16.5	1.29 (d, 6.6)	16.5
7/7′		171.4		171.4
*N*Me-Abu/ *N*Me-Abu′				
19/19′	2.85 (s)	29.5	2.85 (s)	29.5
5/5′	5.36 (m)	54.4	5.37 (m)	54.5
17a/17a′	1.66–1.74 (m)	21.1	1.66–1.74 (m)	21.1
17b/17b′	1.74–1.81 (m)		1.74–1.82 (m)	
18/18′	0.80 (t, 7.2)	9.5	0.80 (t, 7.2)	9.5
4/4′		170.0		170.1
*N*Me-Val/ *N*Me-Val′				
16/16′	2.92 (s)	31.2	2.92 (s)	31.2
2/2′	4.86 (d, 10.8)	62.1	4.87 (d,10.8)	62.1
13/13′	2.28–2.35 (m)	26.3	2.28–2.35 (m)	26.3
14/14′	0.95 (d, 6.6)	19.9	0.96 (d, 6.6)	19.9
15/15′	0.70 (br s)	18.3	0.70 (d, 6.6)	18.3
1/1′		169.3		169.2

^a^: Observed in 2D NMR.

**Table 2:** The HRESIMS/MS fragmentation analysis of Compounds **1** and **2**.

Compound 1					Compound 2				
Meas *m/z*	Calc. *m/z*	mDa	ppm	Formula	Meas *m/z*	Calc. *m/z*	mDa	ppm	Formula
962.4108	962.4137	−2.9	−3.0	C_46_H_57_N_11_NaO_11_	1037.4832	1037.4845	−1.3	−1.3	C_51_H_65_N_12_O_12_
863.3412	863.3453	−4.1	−4.7	C_41_H_48_N_10_NaO_10_	964.3925	964.3929	−0.4	−0.4	C_45_H_55_N_11_NaO_12_
739.4117	739.4143	−2.6	−3.5	C_37_H_55_N_8_O_8_	865.3262	865.3245	1.7	2.0	C_40_H_46_N_10_NaO_11_
640.3446	640.3459	−1.3	−2.0	C_32_H_46_N_7_O_7_	863.3428	863.3453	−2.5	−2.9	C_41_H_48_N_10_NaO_10_
626.3304	626.3302	0.2	0.3	C_31_H_44_N_7_O_7_	642.3270	642.3251	1.9	3.0	C_31_H_44_N_7_O_8_
527.2615	527.2618	−0.3	−0.6	C_26_H_35_N_6_O_6_	628.3102	628.3095	0.7	1.1	C_30_H_42_N_7_O_8_
428.1935	428.1934	0.1	0.2	C_21_H_26_N_5_O_5_	610.2996	610.2989	0.7	1.1	C_30_H_40_N_7_O_7_
414.1781	414.1777	0.4	1.0	C_20_H_24_N_5_O_5_	529.2429	529.2411	1.8	3.4	C_25_H_33_N_6_O_7_
396.1681	396.1672	0.9	2.3	C_20_H_22_N_5_O_4_	527.2623	527.2618	0.5	0.9	C_26_H_35_N_6_O_6_
					511.2306	511.2305	0.1	0.2	C_25_H_31_N_6_O_6_
					428.1939	428.1934	0.5	1.2	C_21_H_26_N_5_O_5_
					416.1592	416.1570	2.2	5.3	C_19_H_22_N_5_O_6_
					414.1778	414.1777	0.1	0.2	C_20_H_24_N_5_O_5_
					398.1472	398.1464	0.8	2.0	C_19_H_20_N_5_O_5_
					396.1679	396.1672	0.7	1.8	C_20_H_22_N_5_O_4_
					380.1367	380.1359	0.8	2.1	C_19_H_18_N_5_O_4_
					337.0923	337.0913	1.0	3.0	C_15_H_14_N_4_NaO_4_

**Table 3:** The UPLC-MS analysis of l-FDLA and d-FDLA derivatives of the hydrolysates of **1**–**2** and amino acid standards.

Samples	l-FDLA (RT; *m/z*)	d-FDLA (RT; *m/z*)	Configuration
l-*N*Me- Ser	8.91 min; 412 [M − H]^−^	9.44 min; 412 [M − H]^−^	
d-Ser	9.74 min; 398 [M − H]^−^	9.11 min; 398 [M − H]^−^	
l-Ala	10.83 min; 382 [M − H]^−^	13.17 min; 382 [M − H]^−^	
l-*N*Me-Abu	13.85 min; 410 [M − H]^−^	15.21 min; 410 [M − H]^−^	
l-*N*Me-Val	15.03 min; 424 [M − H]^−^	17.31 min; 424 [M − H]^−^	
Ser from hydrolysates of **1**	9.74 min; 398 [M − H]^−^	9.11 min; 398 [M − H]^−^	d
Ala from hydrolysates of **1**	10.83 min; 382 [M − H]^−^	13.15 min; 382 [M − H]^−^	l
*N*Me-Abu from hydrolysates of **1**	13.85 min; 410 [M − H]^−^	15.19 min; 410 [M − H]^−^	l
*N*Me-Val from hydrolysates of **1**	15.01 min; 424 [M − H]^−^	17.31 min; 424 [M − H]^−^	l
*N*Me-Ser from hydrolysates of **2**	8.90 min; 412 [M − H]^−^	9.44 min; 412 [M − H]^−^	l
Ser from hydrolysates of **2**	9.75 min; 398 [M − H]^−^	9.11 min; 398 [M − H]^−^	d
Ala from hydrolysates of **2**	10.85 min; 382 [M − H]^−^	13.17 min; 382 [M − H]^−^	l
*N*Me-Abu from hydrolysates of **2**	13.85 min; 410 [M − H]^−^	15.21 min; 410 [M − H]^−^	l
*N*Me-Val from hydrolysates of **2**	15.03 min; 424 [M − H]^−^	17.31 min; 424 [M − H]^−^	l

**Table 4:** The ^1^H (600 MHz, DMSO-*d_6_*) and ^13^C (150 MHz, DMSO-*d_6_*) NMR data of natural- and synthetic-**2**.

Position	Natural-2		Synthetic-2	
	*δ*_H_ (Multi, *J*, Hz)	*δ*_C_	*δ*_H_ (Multi, *J*, Hz)	*δ*_C_
QXA/QXA′				
23/23′		143.1/143.1		143.1/143.1
24/24′	9.51(s)/9.52(s)	143.4/143.4	9.50 (s)/9.52 (s)	143.3/143.3
26/26′	8.14–8.22 (m)	129.1/129.0	8.13–8.24 (m)	129.1/129.0
27/27′	7.98–8.06 (m)	132.1/132.2	7.98–8.06 (m)	132.1/132.2
28/28′	7.98–8.06 (m)	131.5/131.6	7.98–8.06 (m)	131.5/131.6
29/29′	8.14–8.22 (m)	129.4/129.6	8.13–8.24 (m)	129.4/129.6
25a/25a′		143.1/143.1		143.1/143.1
29a/29a′		139.6/139.7		139.6/139.7
22/22′		162.1/162.1		162.2/162.2
Ser/Ser′				
21-NH/21′-NH	8.83 (d, 7.8)/8.56 (d, 7.8)		8.83 (d, 7.8)/8.55 (d, 7.2)	
11/11′	4.90–4.99 (m)/5.04–5.10 (m)	51.0/51.0	4.90–4.99 (m)/5.04–5.10 (m)	51.0/51.0
12a/12′a	4.46–4.54 (m)/4.56–4.60(m)	64.5/65.1^a^	4.46–4.54 (m))/4.55–4.60(m)	64.3/65.0^a^
12b/12′b	4.32–4.38 (d, 10.8)/4.46–4.54 (m)		4.32–4.38 (d, 10.2)/4.46–4.54 (m)	
10/10′		167.9/167.9		167.9/167.9
Ala/Ala′				
9-NH/9′-NH	-		-	
8/8′	4.83–4.88 (m)	45.0/46.4	4.83–4.88 (m)	45.0/46.5
20/20′	1.20 (d, 7.2)/1.42 (d, 7.2)	17.3/18.1	1.20 (d, 6.6)/1.42 (d, 6.6)	17.4/18.1
7/7′		172.1/172.7		172.2 /172.7
*N*Me-Abu				
19	3.27 (s)	30.2	3.28 (s)	30.2
5	5.04–5.10 (m)	54.4	5.04–5.10 (m)	54.4
17a	1.35–1.50 (m)	21.7	1.35–1.50 (m)	21.7
17b	1.62–1.75 (m)		1.62–1.75 (m)	
18	0.64 (m)	10.0	0.64 (m)	10.0
4		169.2		169.2
*N*Me-Ser				
19′	2.69 (s)	29.6	2.69 (s)	29.6
5′	5.27 (d, 7.2)	59.0	5.27 (d, 7.8)	59.0
17a′	3.70–3.74 (m)	59.3	3.70–3.74 (m)	59.3
17b′	3.70–3.74 (m)		3.70–3.74 (m)	
18′-OH	-	-	-	-
4′		168.7		168.7
*N*Me-Val/*N*Me-Val′				
16/16′	2.94 (s)/3.20 (s)	31.2/31.3	2.94 (s)/3.20 (s)	31.2/31.4
2/2′	4.81 (d, 12.0)/4.70 (d, 10.8)	62.1/61.9 ^a^	4.81 (d, 12.6)/4.70 (d, 10.8)	62.0/62.0 ^a^
13/13′	2.38–2.48 (m)/2.10–2.26 (m)	26.4/27.4	2.38–2.48 (m)/2.10–2.26 (m)	26.3/27.4
14/14′	0.94 (d, 6.6)/0.90 (m)	19.9/19.7	0.94 (d, 6.6)/0.90 (d, 6.6)	20.0/19.8
15/15′	0.68 (d, 6.6)/0.75 (m)	18.4/19.0	0.68 (m)/0.75 (m)	18.4/19.1
1/1′		169.4/170.3		169.4/170.4

^a^: Observed in 2D NMR; -: Not determined.

## Data Availability

The sequencing data presented in this study are available in GenBank at NCBI (accession number: MN199475).

## Supplementary Materials

The following supporting information can be downloaded at: https://www.mdpi.com/article/10.3390/md21030143/s1, Figures S1–S10: The HRESIMS, UV, IR, ECD, 1D and 2D NMR spectra of compound **1**; Figure S11: The extracted ion chromatogram (EIC) of l-FDLA and d-FDLA derivatives of amino acid standards. Figures S12–S20: The HRESIMS, UV, IR, ECD, 1D and 2D NMR spectra of compound **2**; Figures S21–S30: The HRESIMS, UV, IR, ^1^H and ^13^C NMR spectra of synthetic-**1** and **2**; Figures S31–S38: Comparison of ^1^H spectra, ^13^C NMR spectra, and UPLC-UV-MS chromatograms of natural and synthetic of **1** and **2**; Table S1: The physicochemical properties of natural and synthetic of **1** and **2**; Tables S2–S3: The antibacterial and cytotoxic activities of synthetic-**1** and synthetic-**2**.
