# Supplementary material for: Isolation, Structure Elucidation, and First Total Synthesis of Quinomycins K and L, Two New Octadepsipeptides from the Maowei Sea Mangrove-Derived Streptomyces sp. B475
Source: Mar Drugs. 2023 Feb 23;21(3):143. doi: 10.3390/md21030143 (PMC10055584; doi:10.3390/md21030143)
Supplement: Supplementary file 1 [file marinedrugs-21-00143-s001.zip › marinedrugs-2238671-supplementary.pdf]

Supporting Information

Isolation, Structure Elucidation, and First Total Synthesis of Quinomycins K and L, Two New Octadepsipeptides from the Maowei Sea Mangrove-derived *Streptomyces* sp. B475

Qin-Pei Lu [1,2,†], Gang Wu [2,†], Xiao-Meng Hao [2], Xin-Xin Hu [3,4], Hao Cai [5], Xiu-Jun Liu [5], Xue-Fu You[3,4], Hong-Wei Guo [1,*], and Cheng-Hang Sun [2,3,6,*]

[1] Guangxi Key Laboratory for Bioactive Molecules Research and Evaluation, Pharmaceutical College, Guangxi Medical University, Nanning 530021, China.

[2] Department of Microbial Chemistry, Institute of Medicinal Biotechnology, Chinese Academy of Medical Sciences & Peking Union Medical College, Beijing 100050, China.

[3] Beijing Key Laboratory of Antimicrobial Agents, Institute of Medicinal Biotechnology, Chinese Academy of Medical Sciences & Peking Union Medical College, Beijing 100050, China.

[4] Department of Pharmacology, Institute of Medicinal Biotechnology, Chinese Academy of Medical Sciences & Peking Union Medical College, Beijing 100050, China.

[5] Department of Oncology, Institute of Medicinal Biotechnology, Chinese Academy of Medical Sciences & Peking Union Medical College, Beijing 100050, China.

[6] College of Eco-Environmental Engineering, Qinghai University, Xining 810016, China.

* Correspondence: hongweiguo@gxmu.edu.cn (H.-W.G.); chenghangsun@hotmail.com or sunchenghang@imb.pumc.edu.cn (C.-H.S.); Tel.: +86-10-63165272

Contents

Figure S1. The HRESIMS of compound **1**.
Figure S2. The UV spectrum of compound **1**.
Figure S3. The IR spectrum of compound **1**.
Figure S4. The ECD spectrum of compound **1**.
Figure S5. The ^{1}H NMR (600 MHz) spectrum of compound **1** in DMSO-d_6.
Figure S6. The ^{13}C NMR (150 MHz) spectrum of compound **1** in DMSO-d_6.
Figure S7. The ^{1}H-^{1}H COSY spectrum of compound **1**.
Figure S8. The HSQC spectrum of compound **1**.
Figure S9. The HMBC spectrum of compound **1**.
Figure S10. The ROESY spectrum of compound **1**.
Figure S11. The Extracted Ion Chromatogram (EIC) of L-FDLA and D-FDLA derivatives of amino acid standards.
Figure S12. The HRESIMS of compound **2**.
Figure S13. The UV spectrum of compound **2**.
Figure S14. The IR spectrum of compound **2**.
Figure S15. The ECD spectrum of compound **2**.
Figure S16. The ^{1}H NMR (600 MHz) spectrum of compound **2** in DMSO-d_6.
Figure S17. The ^{13}C NMR (150 MHz) spectrum of compound **2** in DMSO-d_6.
Figure S18. The ^{1}H-^{1}H COSY spectrum of compound **2**.
Figure S19. The HSQC spectrum of compound **2**.
Figure S20. The HMBC spectrum of compound **2**.
Figure S21. The HRESIMS of synthetic-**1**.
Figure S22. The UV spectrum of synthetic-**1**.
Figure S23. The IR spectrum of synthetic-**1**.
Figure S24. The ^{1}H NMR (600 MHz) spectrum of synthetic-**1** in DMSO-d_6.
Figure S25.1 The ^{13}C NMR (150 MHz) spectrum of synthetic-**1** in DMSO-d_6.
Figure S26. The HRESIMS of synthetic-**2**.
Figure S27. The UV spectrum of synthetic-**2**.
Figure S28.2 The IR spectrum of synthetic-**2**.
Figure S29.3 The ^{1}H NMR (600 MHz) spectrum of synthetic-**2** in DMSO-d_6.
Figure S30. The ^{13}C NMR (150 MHz) spectrum of synthetic-**2** in DMSO-d_6.
Figure S31. Comparison of ^{1}H NMR spectra (600 MHz) of natural and synthetic of **1**.
Figure S32. Comparison of ^{13}C NMR spectra (150 MHz) of natural and synthetic of **1**.
Figure S33. Comparison of ^{1}H NMR spectra (600 MHz) of natural and synthetic of **2**.
Figure S34. Comparison of ^{13}C NMR spectra (125 MHz) of natural and synthetic of **2**.
Figure S35. The UPLC-UV-MS chromatograms of **1** (natural, synthetic, and co-elution) in the condition of ACN/water with 0.1% formic acid.
Figure S36. The UPLC-UV-MS chromatograms of **1** (natural, synthetic, and co-elution) in the condition of MeOH/water with 0.1% formic acid.
Figure S37. The UPLC-UV-MS chromatograms of **2** (natural, synthetic, and co-elution) in the condition of ACN/ water with 0.1% formic acid.
Figure S38. The UPLC-UV-MS chromatograms of **2** (natural, synthetic, and co-elution) in the condition of MeOH/ water with 0.1% formic acid.

Table S1. The physicochemical properties of natural-**1**, synthetic-**1**, natural-**2**, and synthetic-**2**.

Table S2. The antibacterial activities of synthetic-**1** and synthetic-**2** (MIC, μg/mL).

Table S3. The cytotoxic activity of synthetic-**1** and synthetic-**2** against H460 lung cancer cells.

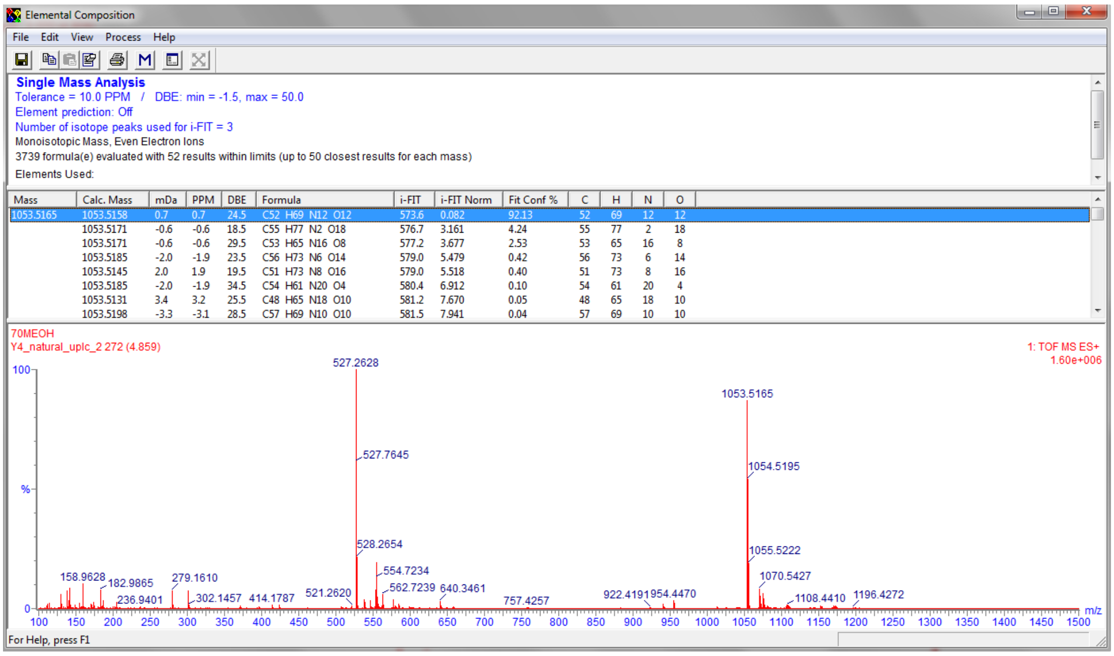

Mass	Calc. Mass	mDa	PPM	DBE	Formula	i-FIT	i-FIT Norm	Fit Conf %	C	H	N	O
1053.5165	1053.5158	0.7	0.7	24.5	C52 H69 N12 O12	573.6	0.082	92.13	52	69	12	12
	1053.5171	-0.6	-0.6	18.5	C55 H77 N2 O18	576.7	3.161	4.24	55	77	2	18
	1053.5171	-0.6	-0.6	29.5	C53 H65 N16 O8	577.2	3.677	2.53	53	65	16	8
	1053.5185	-2.0	-1.9	23.5	C56 H73 N6 O14	579.0	5.479	0.42	56	73	6	14
	1053.5145	2.0	1.9	19.5	C51 H73 N8 O16	579.0	5.518	0.40	51	73	8	16
	1053.5185	-2.0	-1.9	34.5	C54 H61 N20 O4	580.4	6.912	0.10	54	61	20	4
	1053.5131	3.4	3.2	25.5	C48 H65 N18 O10	581.2	7.670	0.05	48	65	18	10
	1053.5198	-3.3	-3.1	28.5	C57 H69 N10 O10	581.5	7.941	0.04	57	69	10	10

Figure S1. The HRESIMS of compound **1**.

光谱峰值检测报告

2020-11-11 11:55:06

数据集: Y4_2 - RawData

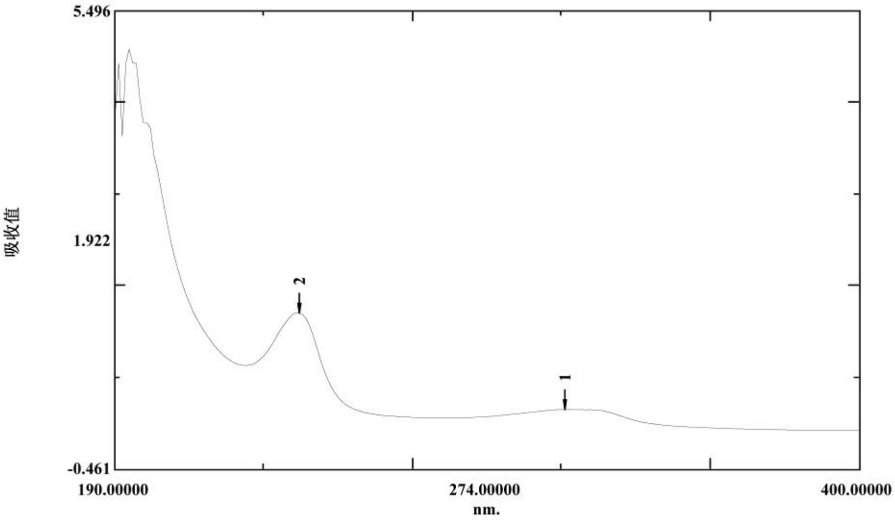

测定属性
波长范围 (nm.): 190.00000 到 400.00000
扫描速度: 中速
采样间隔: 1.0
自动采样间隔: 停用
扫描模式: 自动

No.	P/V	波长(nm)	吸收值
1	↑	317.00000	0.307
2	↑	242.00000	1.566
3	↓	284.00000	0.195
4	↓	227.00000	0.880

仪器属性
仪器类型: UV-2500PC 系列
测定方式: 吸收值
狭缝宽: 2.0 nm
光源转换波长: 350.0 nm
S/R 转换: 标准

附件属性
附件: 6联池
池数目: 1

样品准备属性
重量: 0.03MG
体积: 1.5ML
稀释:
光程长: 10
附加信息: mg/ml

Figure S2. The UV spectrum of compound **1**.

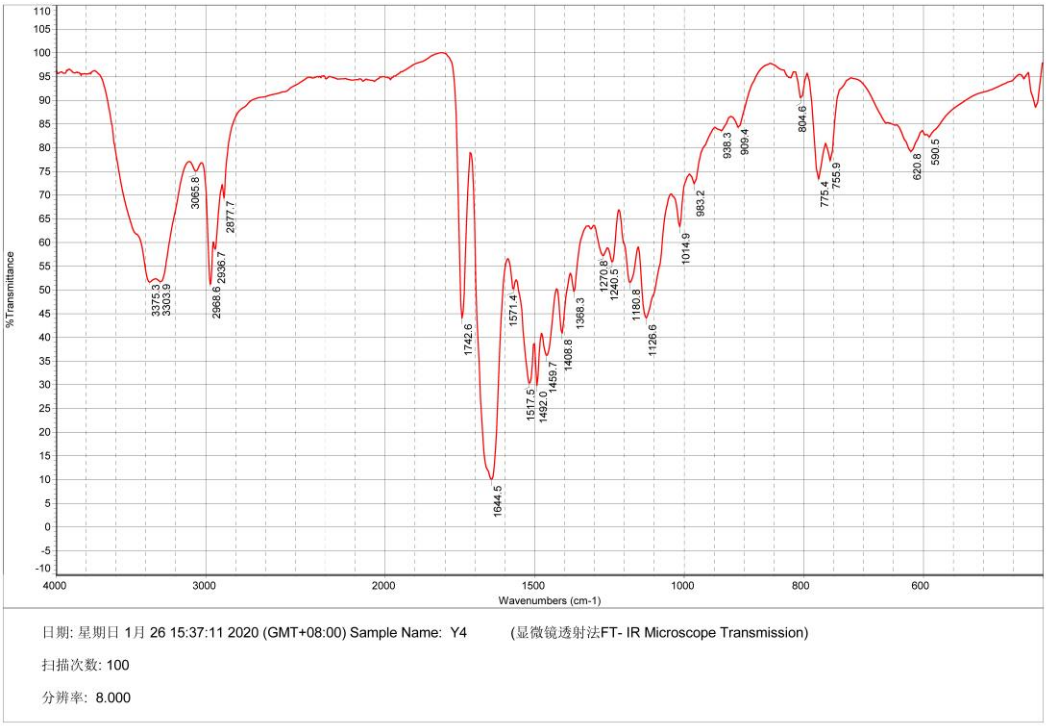

Figure S3. The IR spectrum of compound **1**.

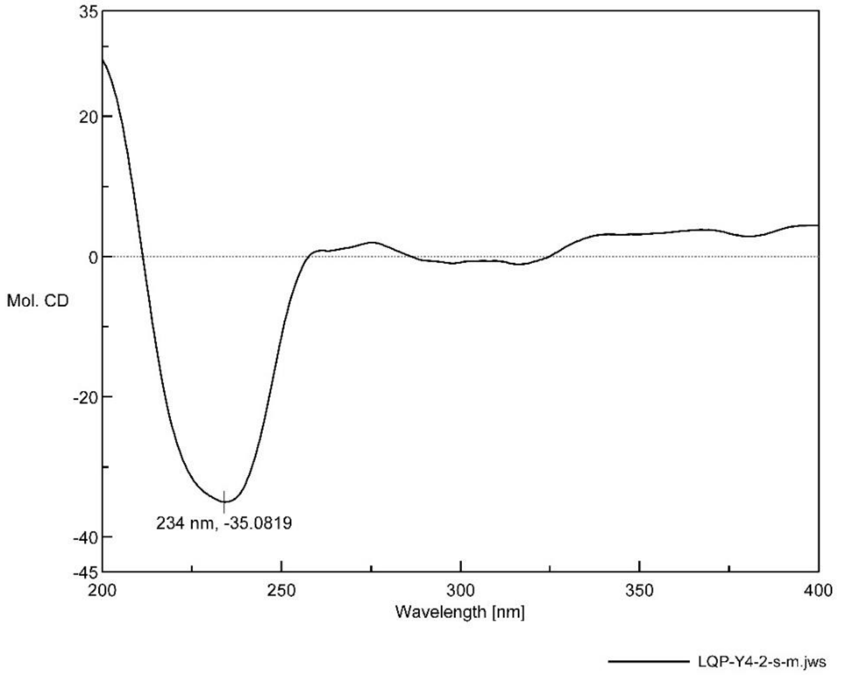

[Measurement Information]

Instrument Name	J-815
Model Name	J-815
Serial No.	A024461168
Accessory	Standard
Accessory S/N	A024461168
Cell Length	1 mm
Measurement date	2020/11/11 15:28
Photometric Mode	CD, HT, Abs
Measure Range	400 - 200 nm
Data pitch	0.5 nm
Sensitivity	Standard
D.I.T.	1 sec
Bandwidth	1.00 nm
Start Mode	Immediately
Scanning Speed	100 nm/min
Baseline Correction	Baseline
Shutter Control	Auto
CD Detector	PMT
PMT Voltage	Auto
Accumulations	2
Solvent	MEOH
Concentration	0.02 (w/v)%

Figure S4. The ECD spectrum of compound **1**.

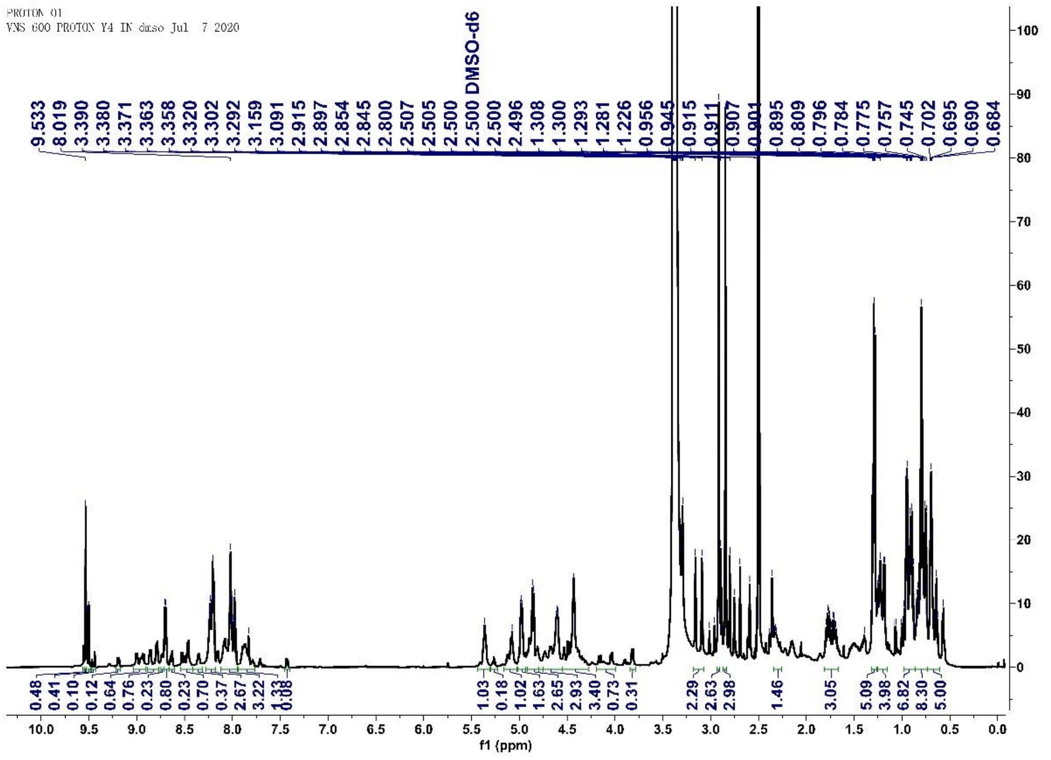

Figure S5. The ^{1}H NMR (600 MHz) spectrum of compound **1** in DMSO-d_6.

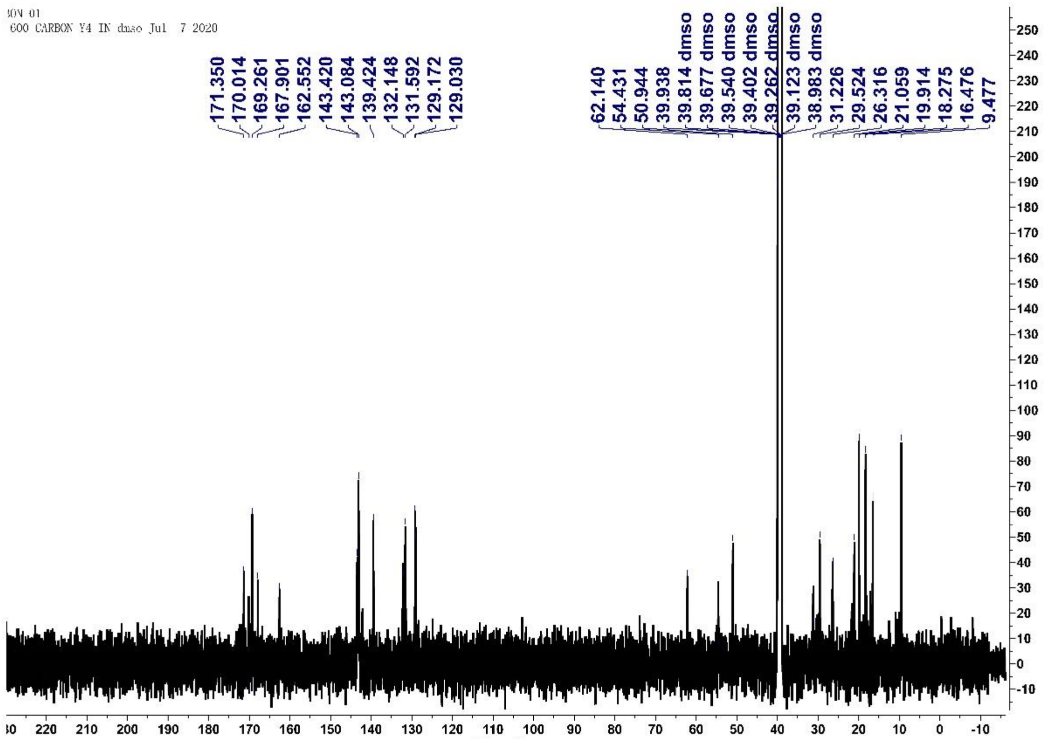

Figure S6. The ^{13}C NMR (150 MHz) spectrum of compound **1** in DMSO-d_6.

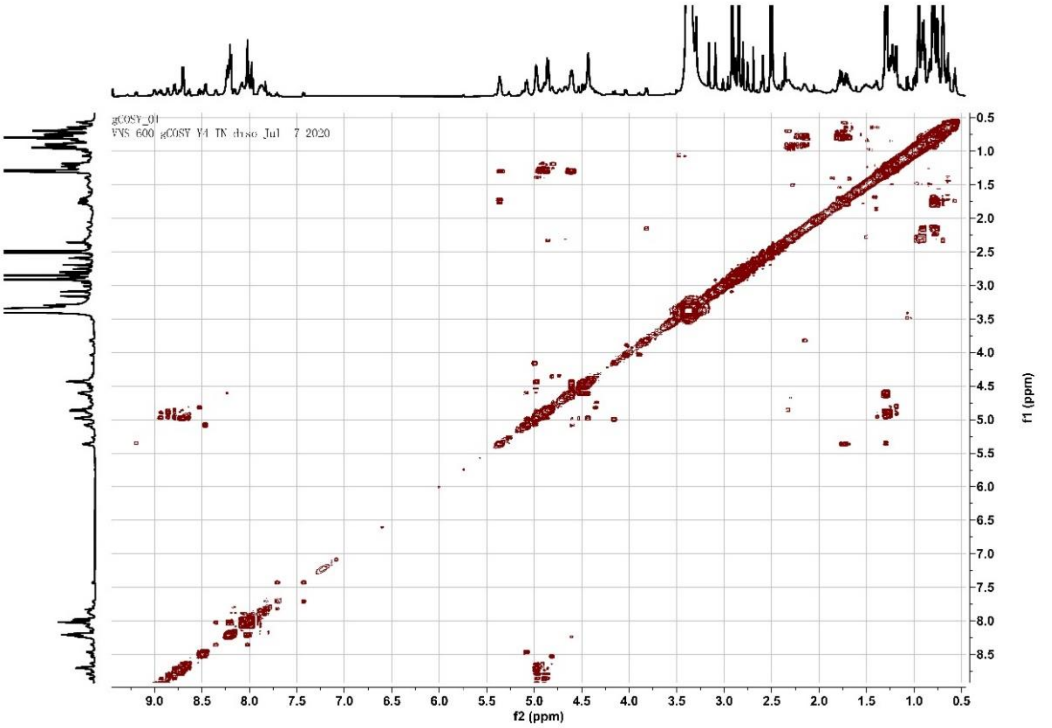

Figure S7. The ^{1}H-^{1}H COSY spectrum of compound **1**.

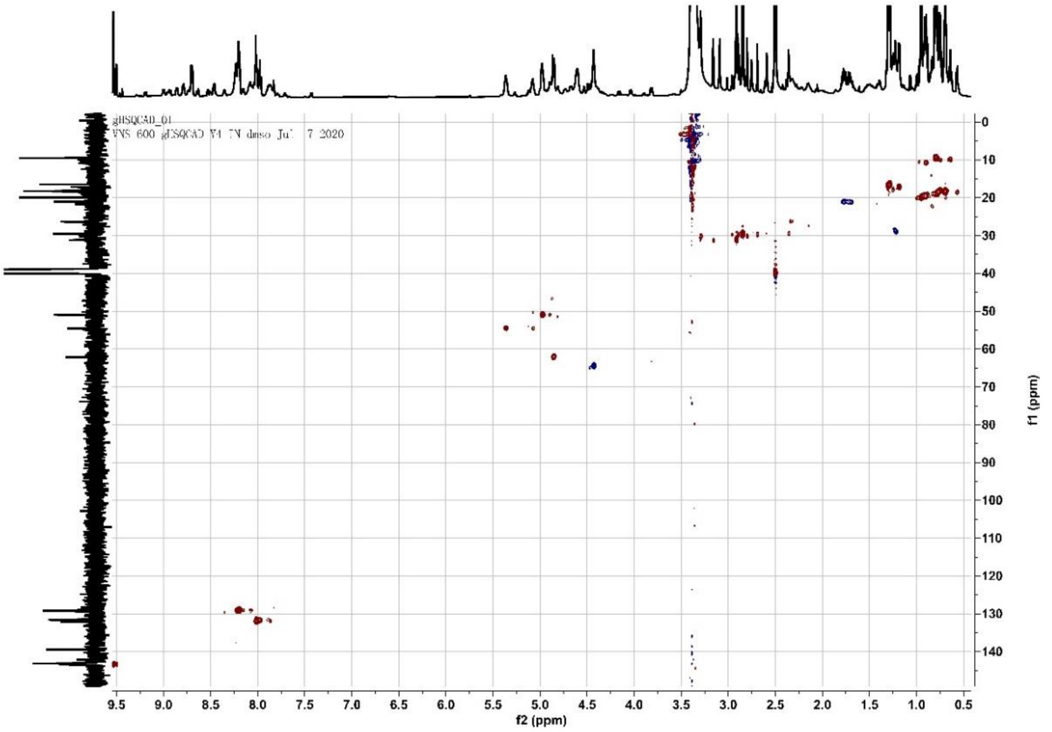

Figure S8. The HSQC spectrum of compound **1**.

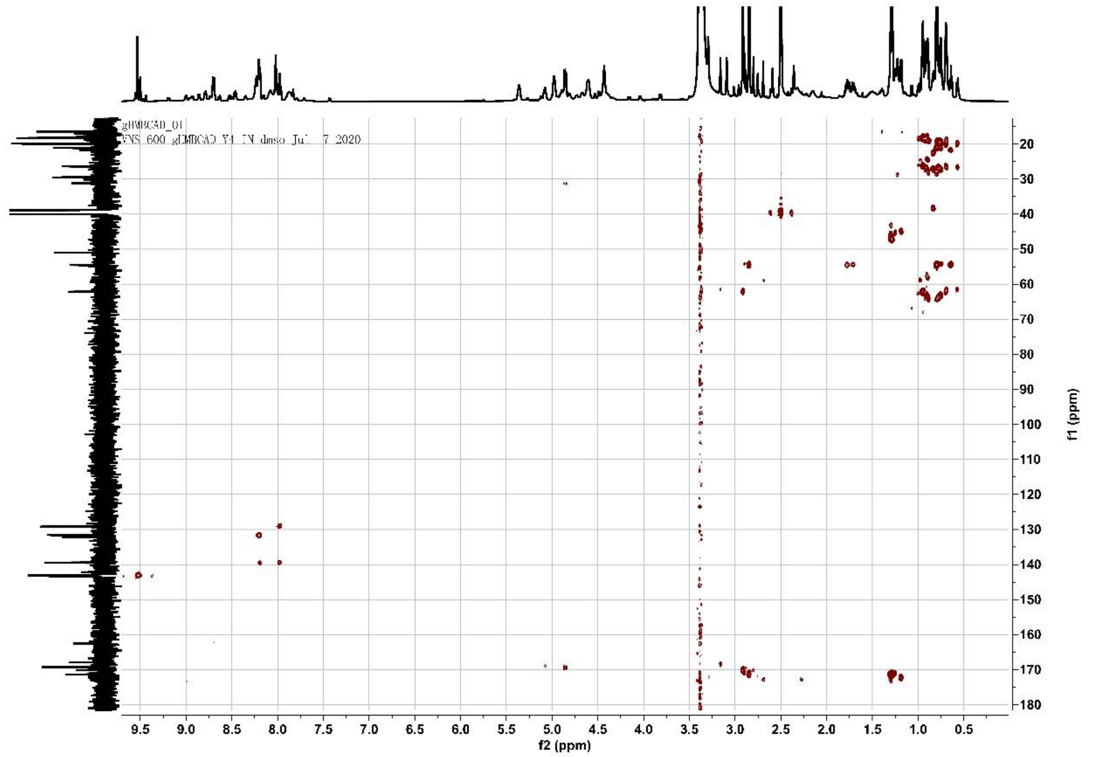

Figure S9. The HMBC spectrum of compound **1**.

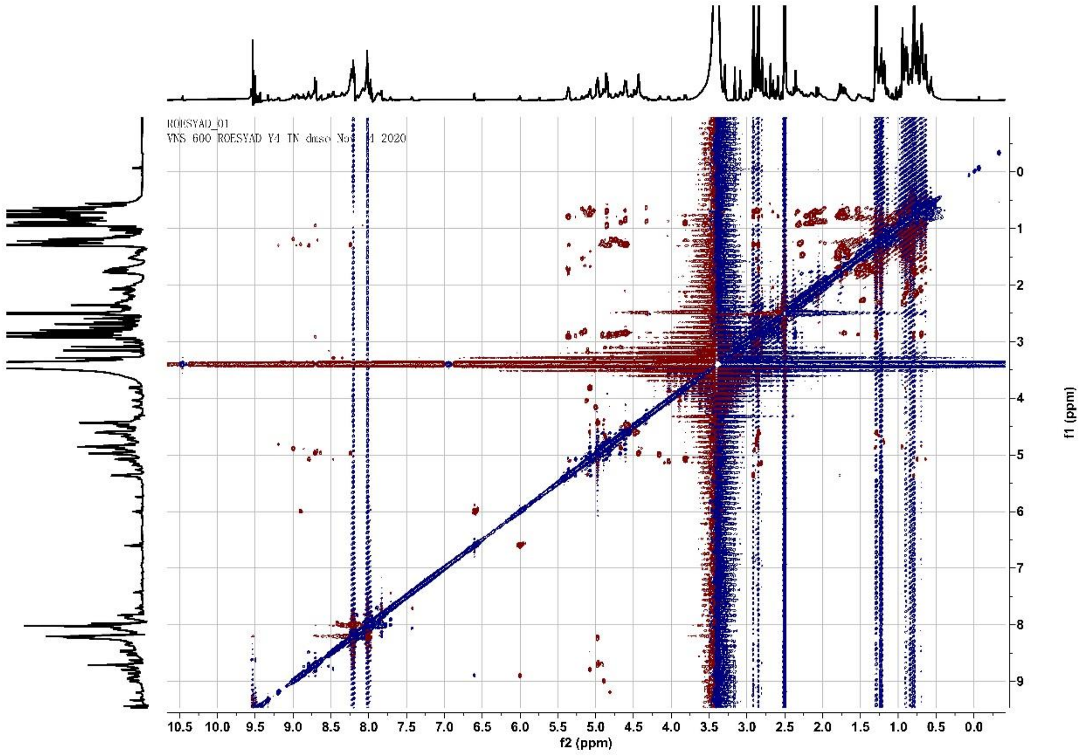

Figure S10. The ROESY spectrum of compound **1**.

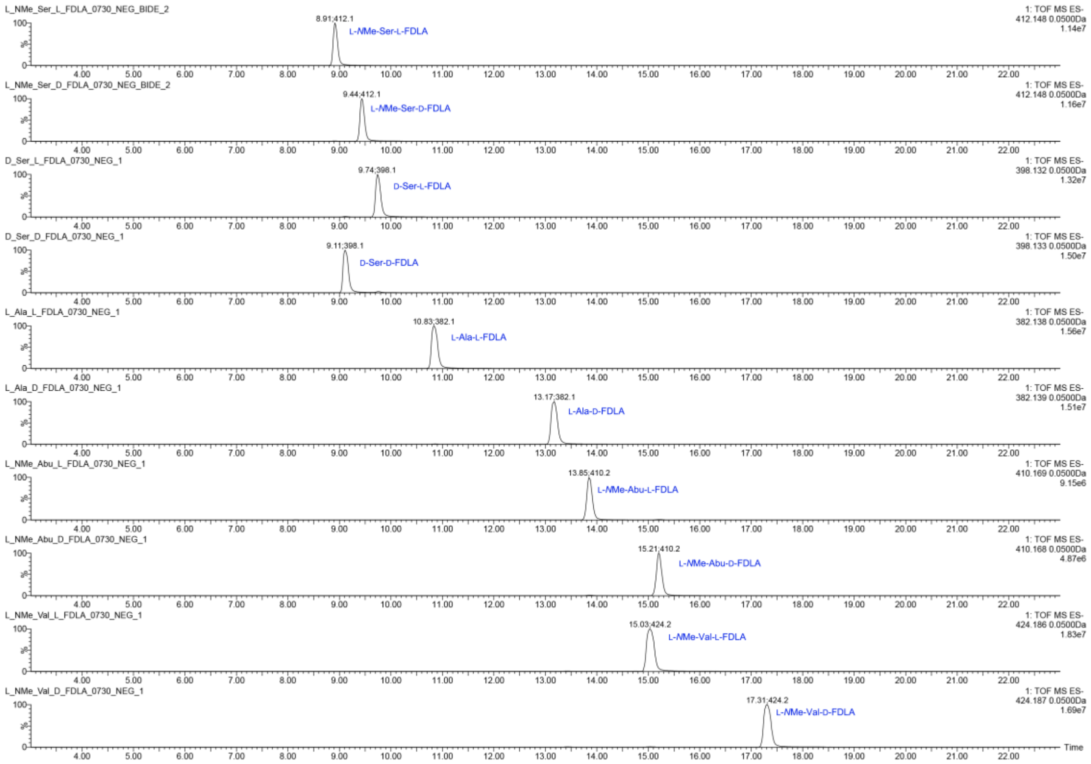

Figure S11. The Extracted Ion Chromatogram (EIC) of L-FDLA and D-FDLA derivatives of amino acid standards.

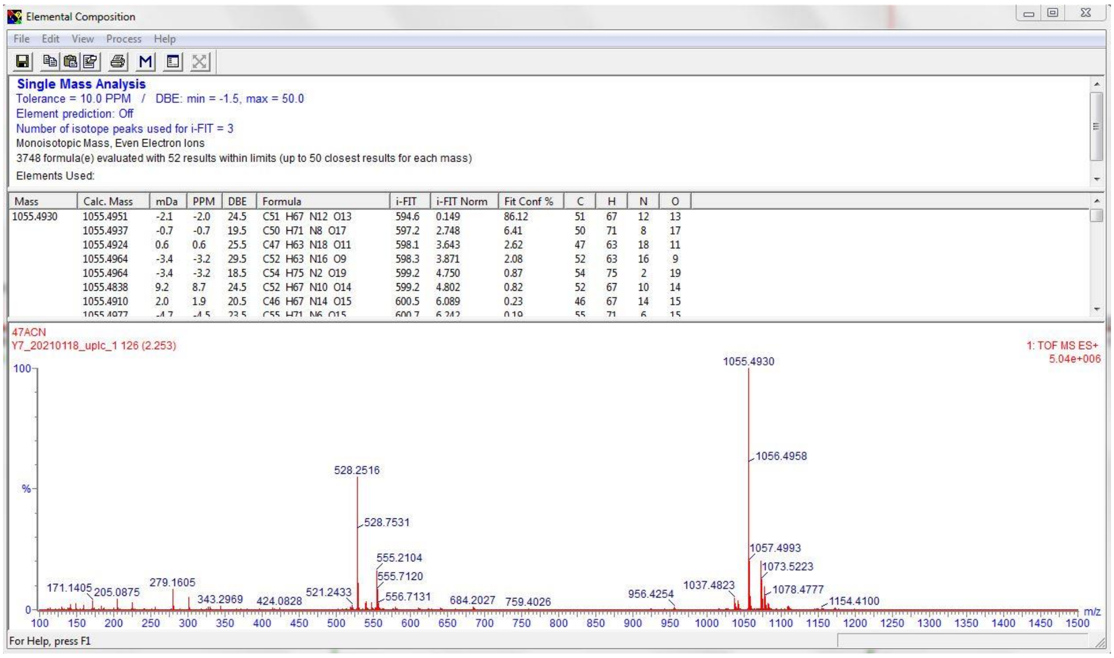

Figure S12. The HRESIMS of compound **2**.

光谱峰值检测报告

2012-06-13 00:04:39

数据集: Y7_20210111 - RawData

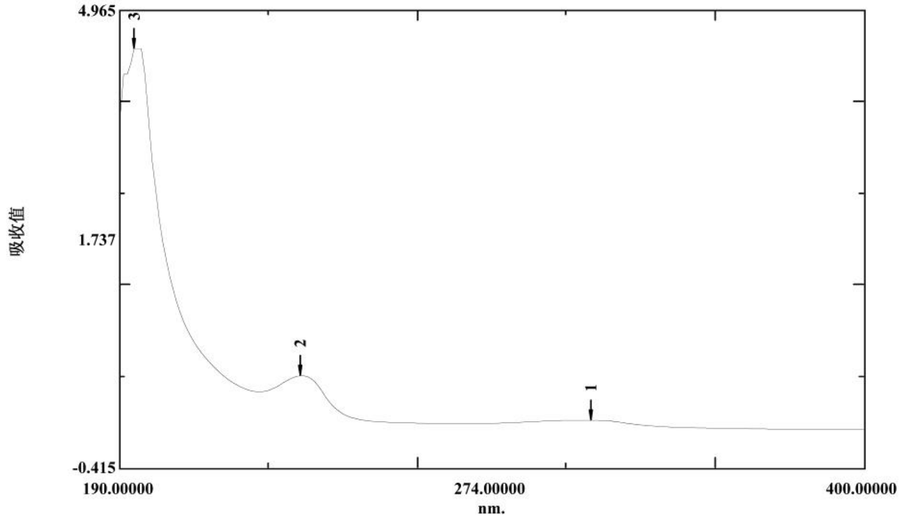

测定属性
波长范围 (nm.): 190.00000 到 400.00000
扫描速度: 中速
采样间隔: 1.0
自动采样间隔: 停用
扫描模式: 自动

仪器属性
仪器类型: UV-2500PC 系列
测定方式: 吸收值
狭缝宽: 2.0 nm
光源转换波长: 350.0 nm
S/R 转换: 标准

附件属性
附件: 6联池
池数目: 1

样品准备属性
重量: 0.02
体积: 1
稀释:
光程长: 10
附加信息: 0.018mg/ml

No.	P/V	波长(nm)	吸收值
1	↑	323.00000	0.141
2	↑	241.00000	0.666
3	↑	194.00000	4.516
4	↓	288.00000	0.100
5	↓	229.00000	0.477

Figure S13. The UV spectrum of compound **2**.

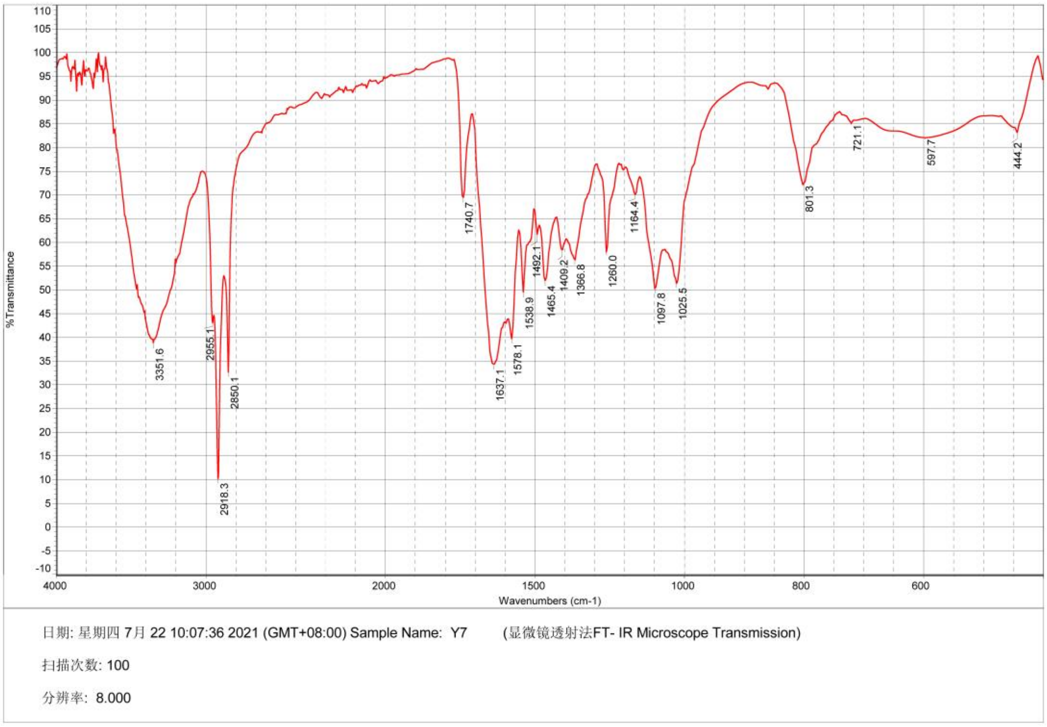

Figure S14. The IR spectrum of compound **2**.

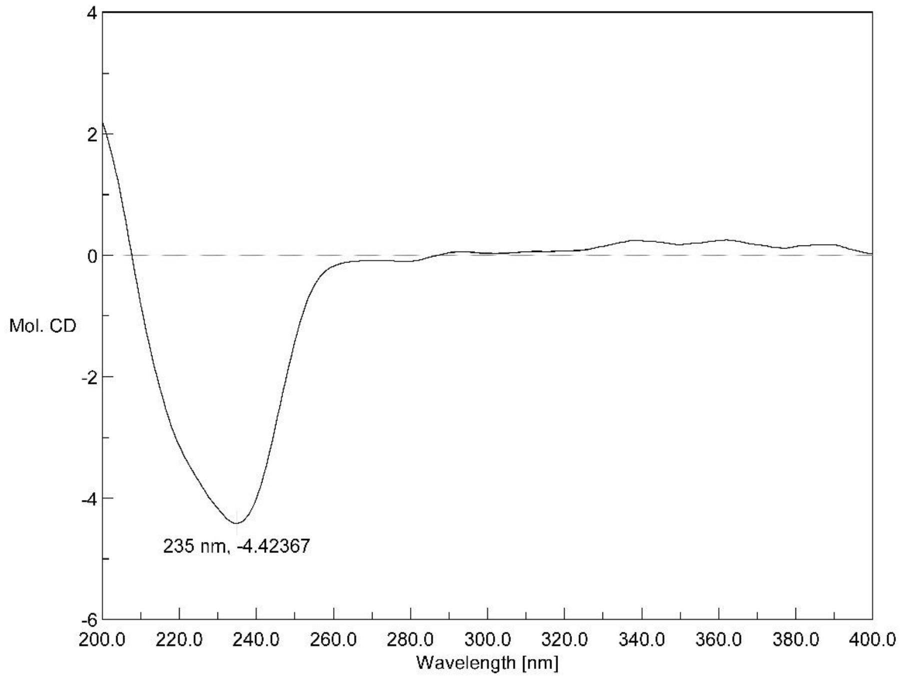

[Comments]
Sample name Y7
Comment
[Measurement Information]
Instrument Name IMM-CD
Model Name J-815
Serial No. A024461168

Accessory Standard
Accessory S/N A005461185
Cell Length 1 mm

Measurement date 2021-01-11 11:25

Photometric Mode CD, HT, Abs
Measure Range 400 - 200 nm
Data pitch 0.5 nm
Sensitivity Standard
D.I.T. 1 sec
Bandwidth 1.00 nm
Start Mode Immediately
Scanning Speed 100 nm/min
Baseline Correction Baseline
Shutter Control Auto
CD Detector PMT
PMT Voltage Auto
Accumulations 2
Solvent MEOH
Concentration 0.018 (w/v)%

——— Y7-1-s-m.jws

[Detailed Information]
Creation date 2021-01-12 13:53

Data array type Linear data array * 3
Horizontal axis Wavelength [nm]
Vertical axis(1) Mol. CD
Vertical axis(2) HT [V]
Vertical axis(3) Abs
Start 400 nm
End 200 nm
Data interval 0.5 nm
Data points 401

Figure S15. The ECD spectrum of compound **2**.

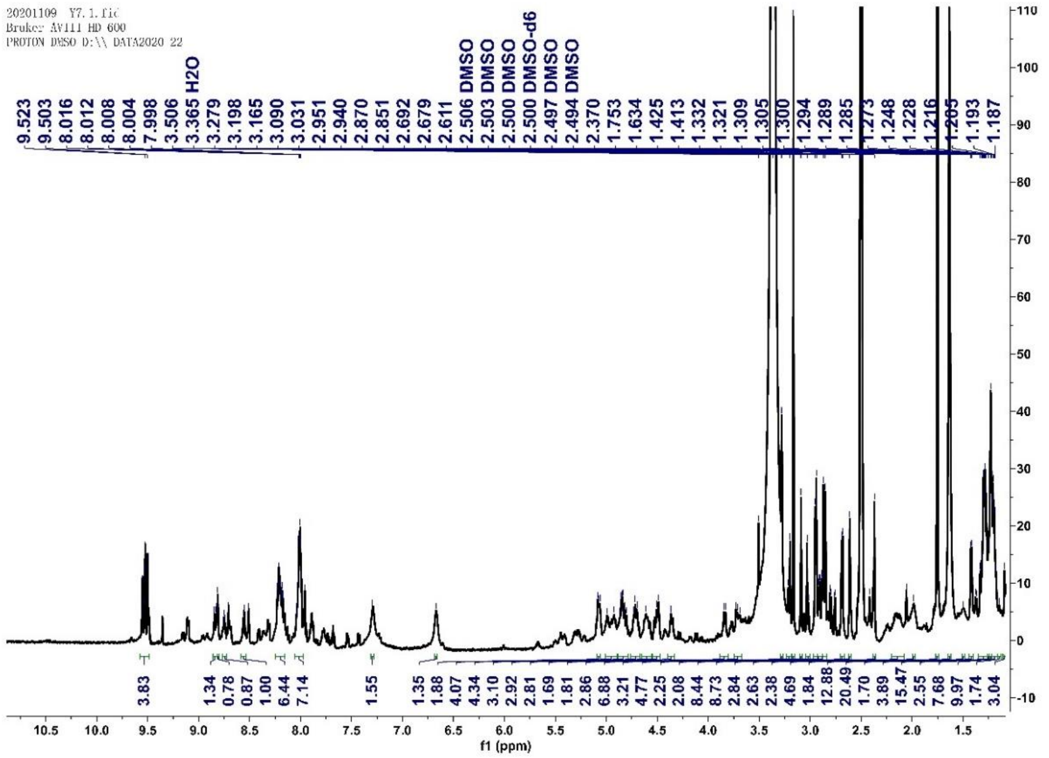

Figure S16. The ^{1}H NMR (600 MHz) spectrum of compound **2** in DMSO-d_6.

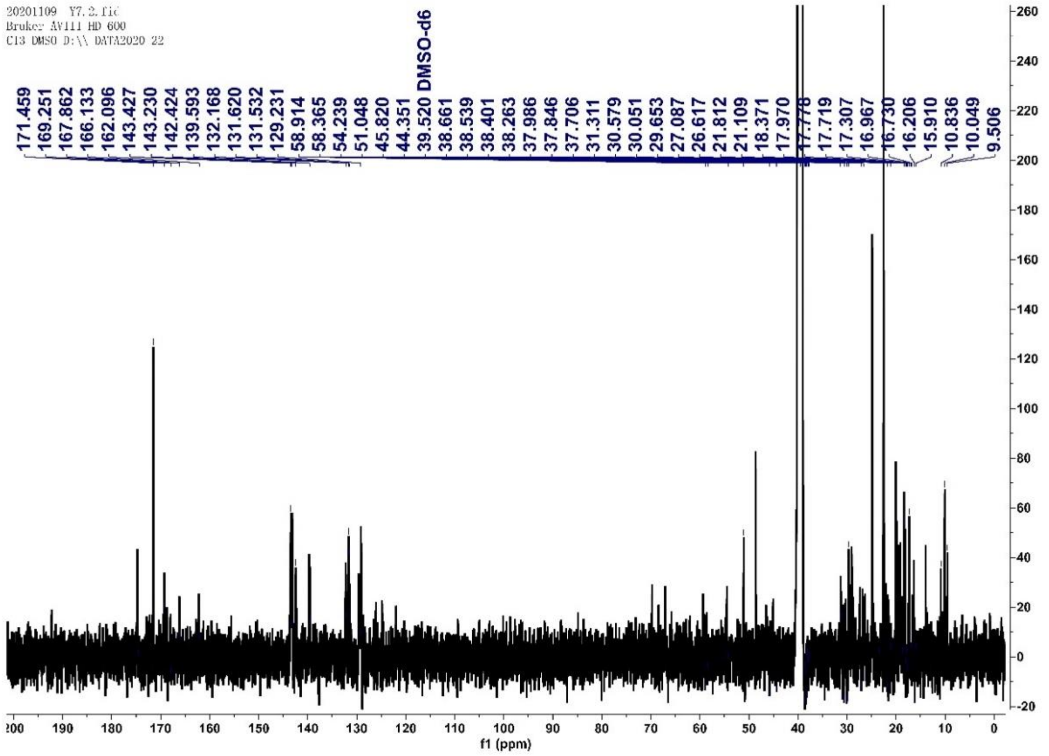

Figure S17. The ^{13}C NMR (150 MHz) spectrum of compound **2** in DMSO-d_6.

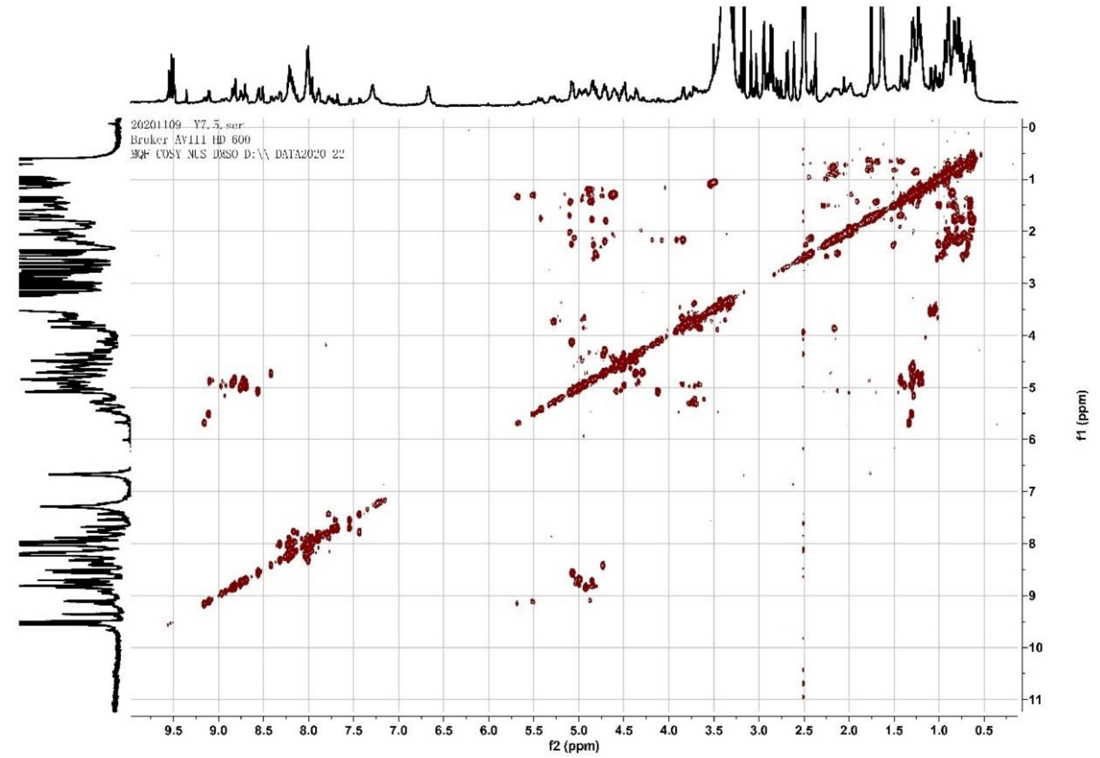

Figure S18. The ^1H-^1H COSY spectrum of compound **2**.

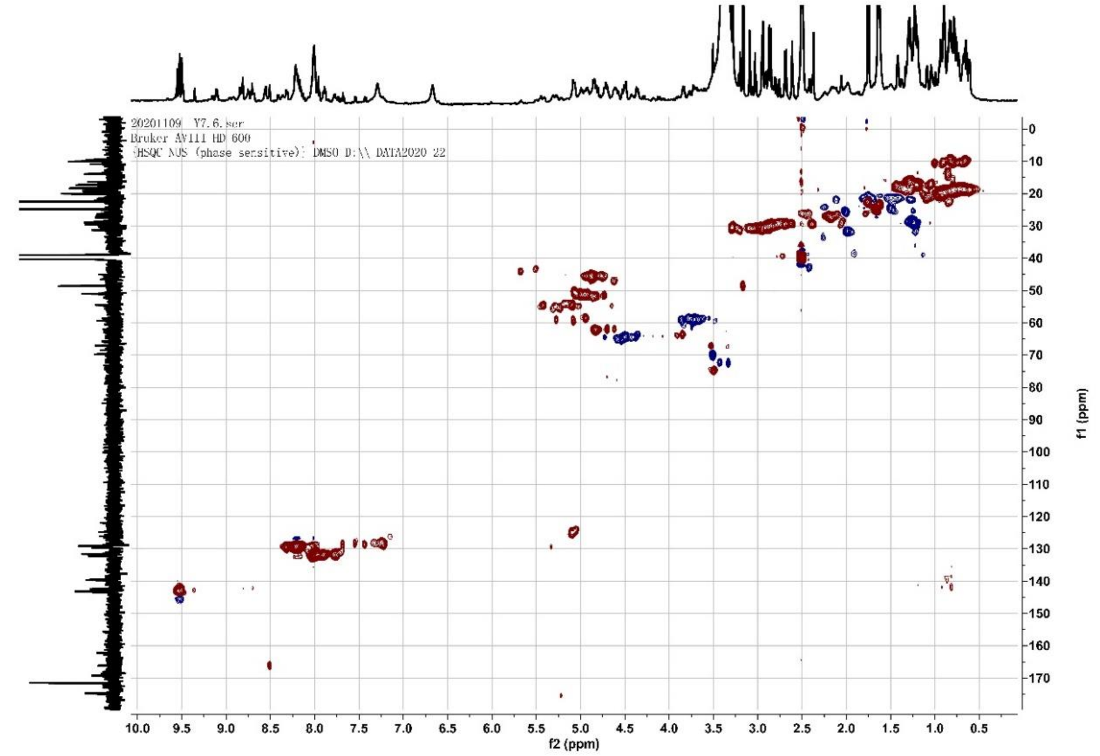

Figure S19. The HSQC spectrum of compound **2**.

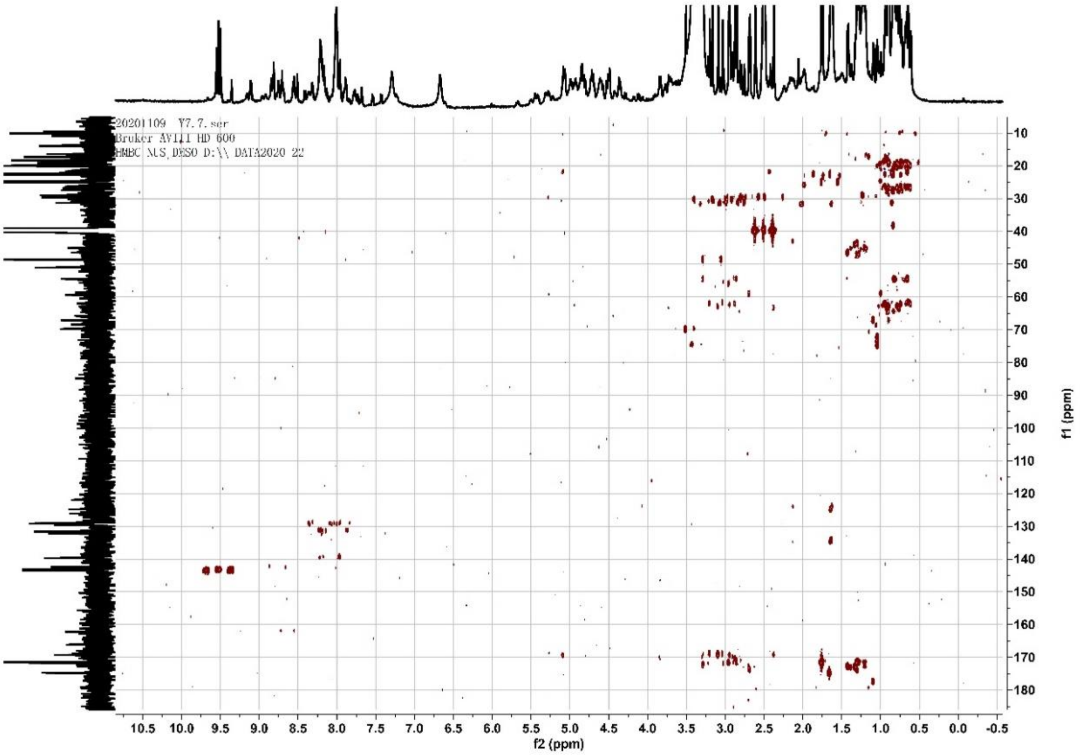

Figure S20. The HMBC spectrum of compound **2**.

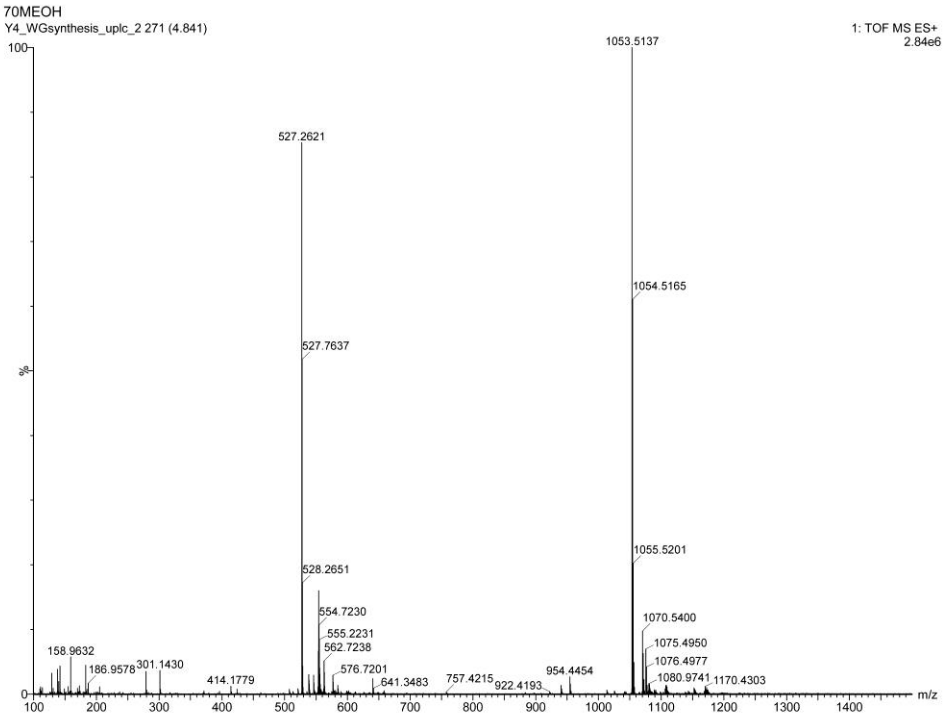

Figure S21. The HRESIMS of synthetic-**1**.

光谱峰值检测报告

2012-08-21 02:42:06

数据集: Y4s_20210111 - RawData

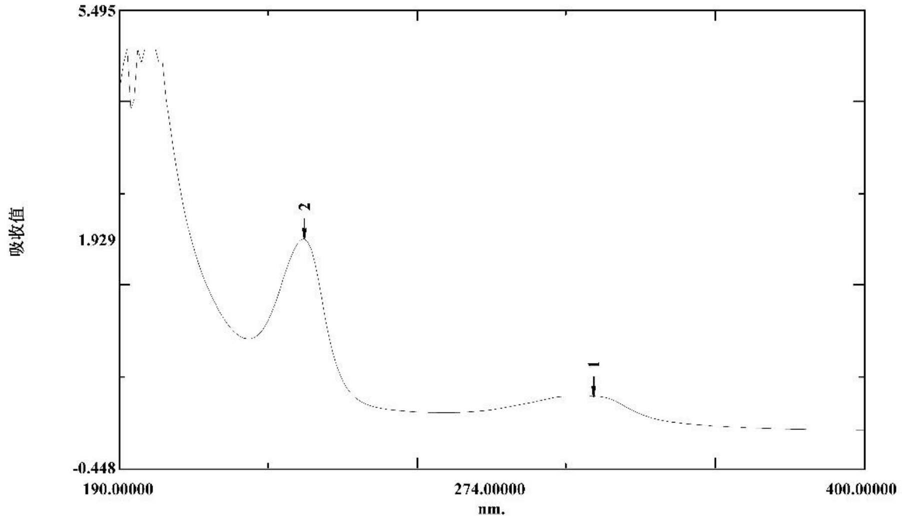

测定属性	
波长范围 (nm.):	190.00000 到 400.00000
扫描速度:	中速
采样间隔:	1.0
自动采样间隔:	停用
扫描模式:	自动

No.	P/V	波长(nm)	吸收值
1	↑	324.00000	0.484
2	↑	242.00000	2.533
3	↓	282.00000	0.269
4	↓	226.00000	1.232

仪器属性	
仪器类型:	UV-2500PC 系列
测定方式:	吸收值
狭缝宽:	2.0 nm
光源转换波长:	350.0 nm
S/R 转换:	标准

附件属性	
附件:	6联池
池数目:	1

样品准备属性	
重量:	0.02
体积:	1
稀释:	
光程长:	10
附加信息:	0.04mg/ml

Figure S22. The UV spectrum of synthetic-**1**.

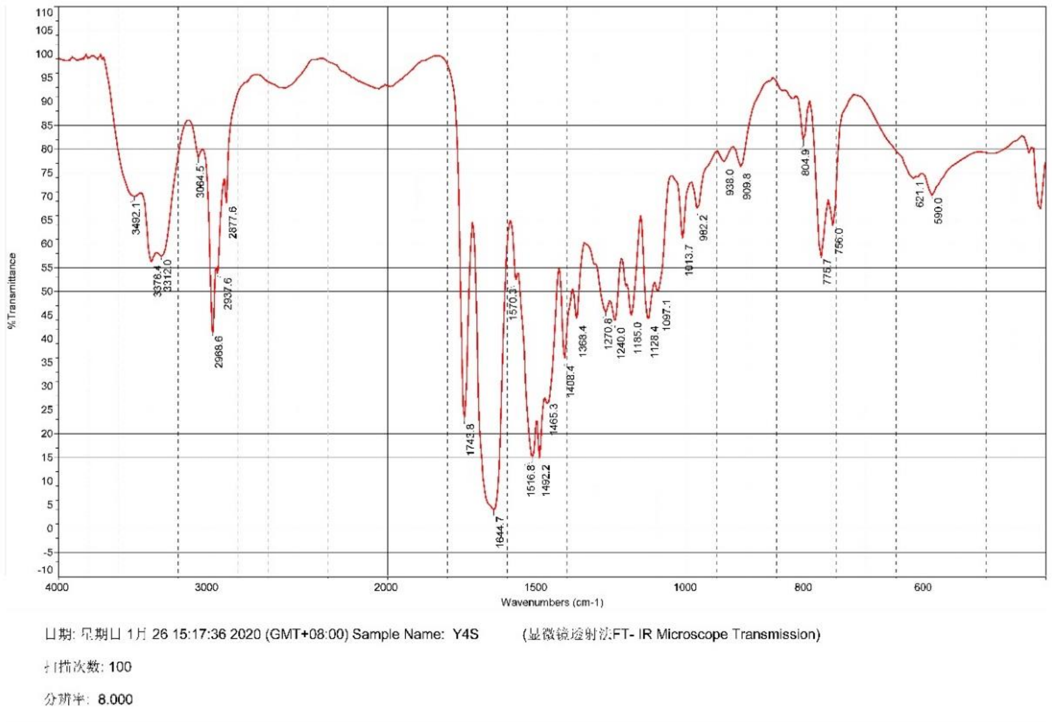

Figure S23. The IR spectrum of synthetic-**1**.

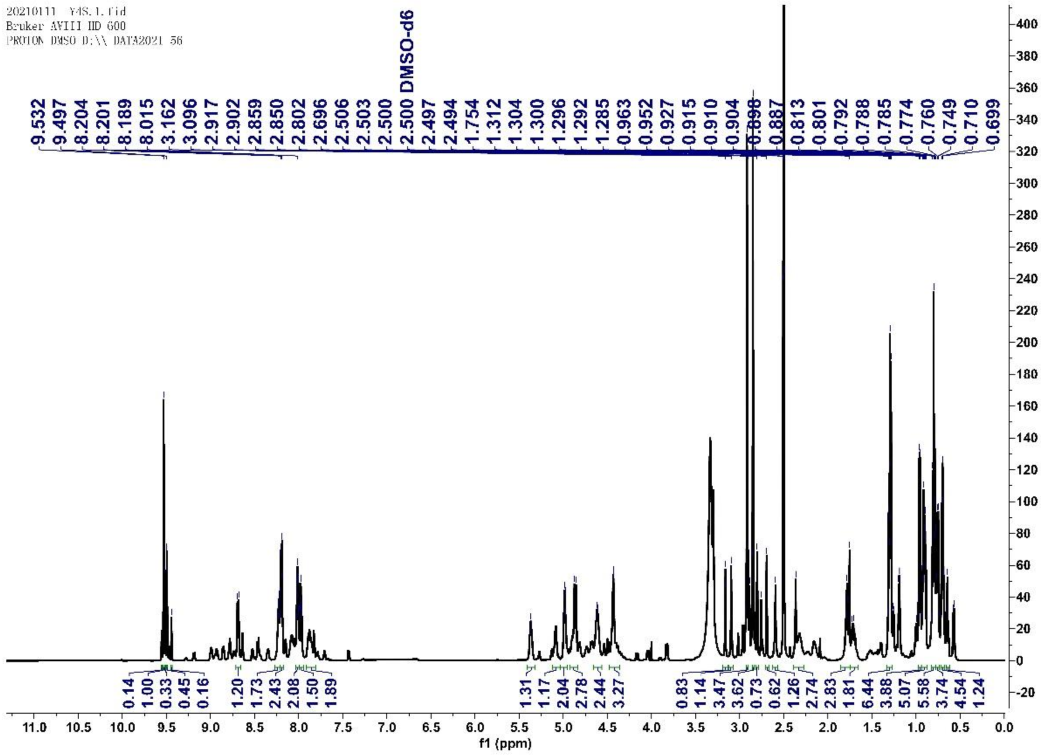

Figure S24. The ^{1}H NMR (600 MHz) spectrum of synthetic-**1** in DMSO-d_6.

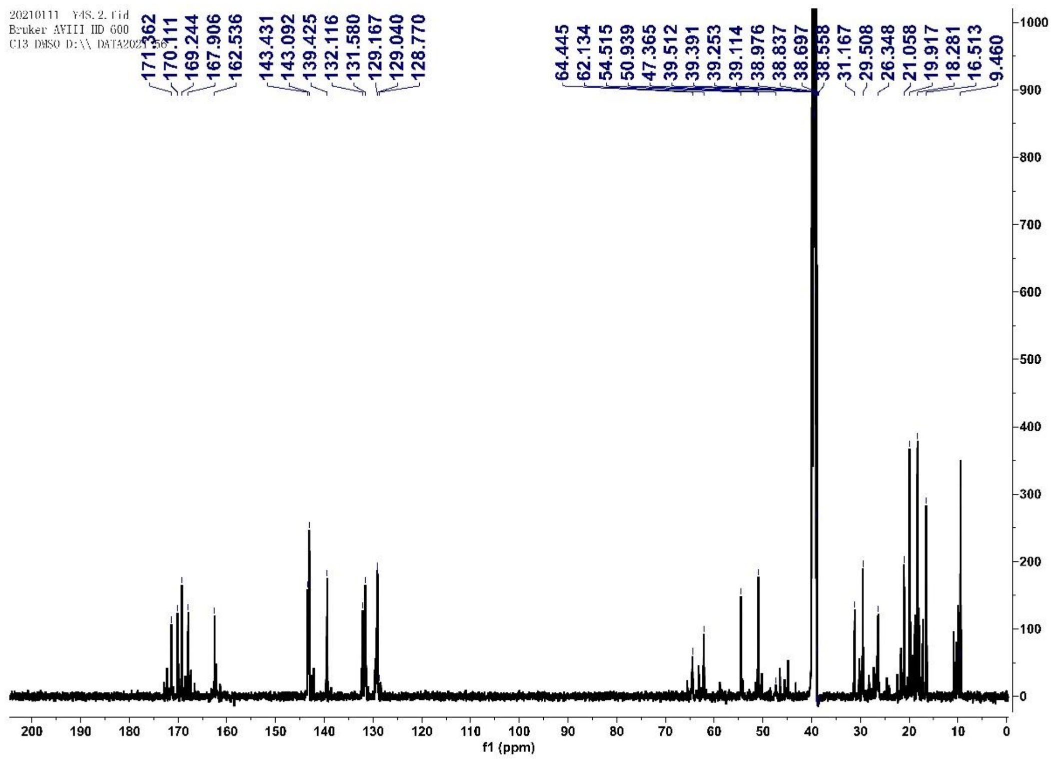

Figure S25.4 The ^{13}C NMR (150 MHz) spectrum of synthetic-**1** in DMSO-d_6.

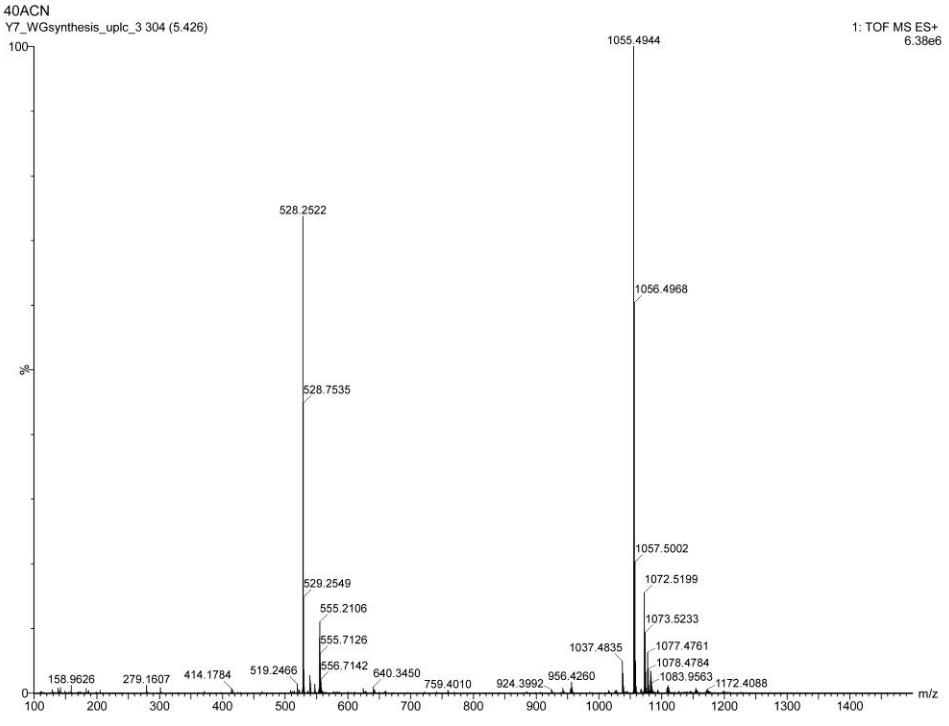

Figure S26. The HRESIMS of synthetic-**2**.

光谱峰值检测报告

2012-08-21　02:46:23

数据集:　Y7S_gaonongdu20210111 - RawData

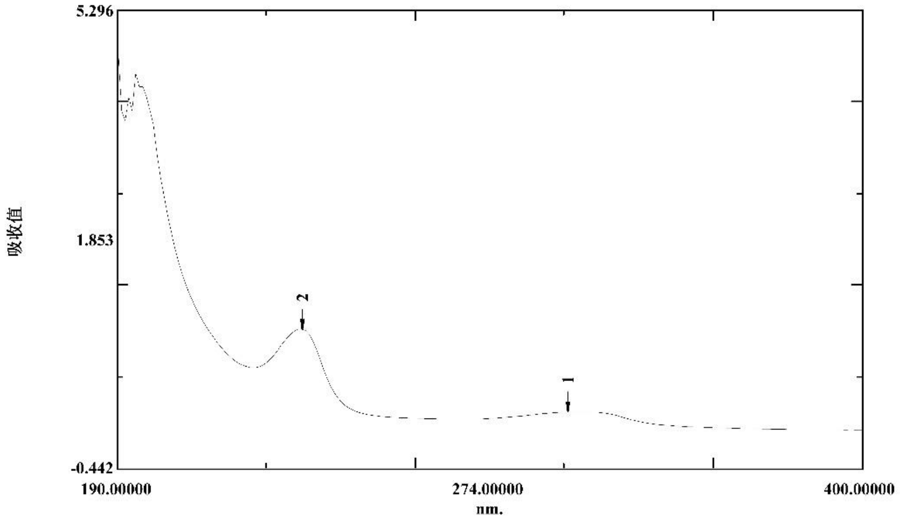

测定属性
波长范围 (nm.):　190.00000 到 400.00000
扫描速度:　中速
采样间隔:　1.0
自动采样间隔:　停用
扫描模式:　自动

No.	P/V	波长(nm)	吸收值
1	↑	317.00000	0.258
2	↑	242.00000	1.302
3	↓	287.00000	0.169
4	↓	228.00000	0.816

仪器属性
仪器类型:　UV-2500PC 系列
测定方式:　吸收值
狭缝宽:　2.0 nm
光源转换波长:　350.0 nm
S/R 转换:　标准

附件属性
附件:　6联池
池数目:　1

样品准备属性
重量:　0.02
体积:　1
稀释:
光程长:　10
附加信息:　0.04mg/ml

Figure S27. The UV spectrum of synthetic-**2**.

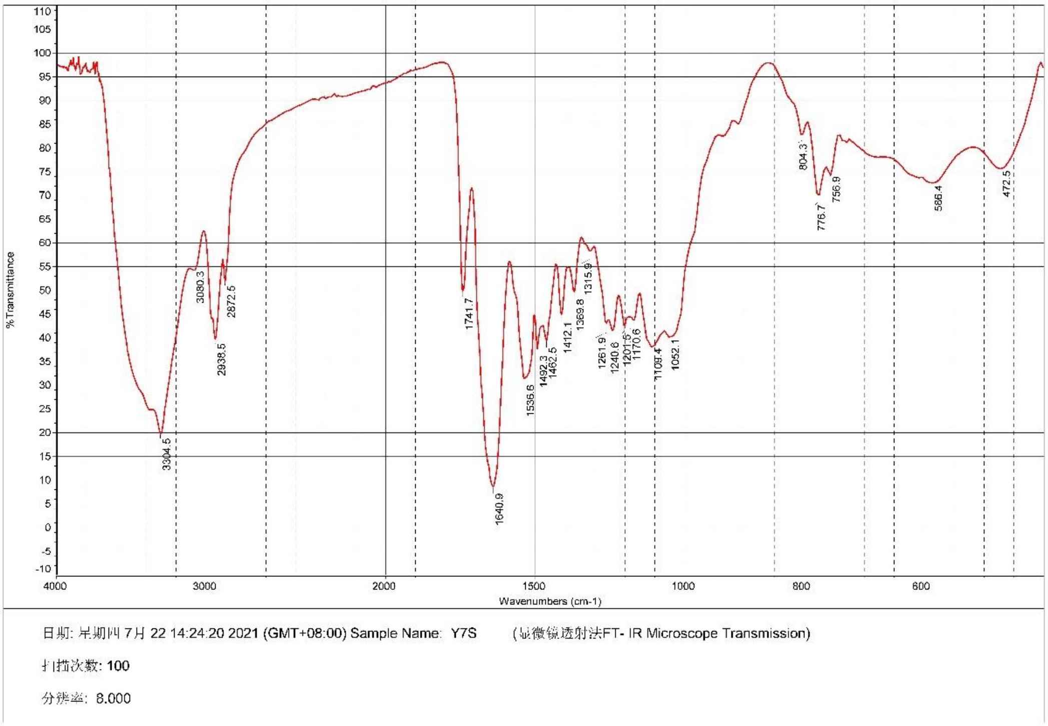

Figure S28.5 The IR spectrum of synthetic-**2**.

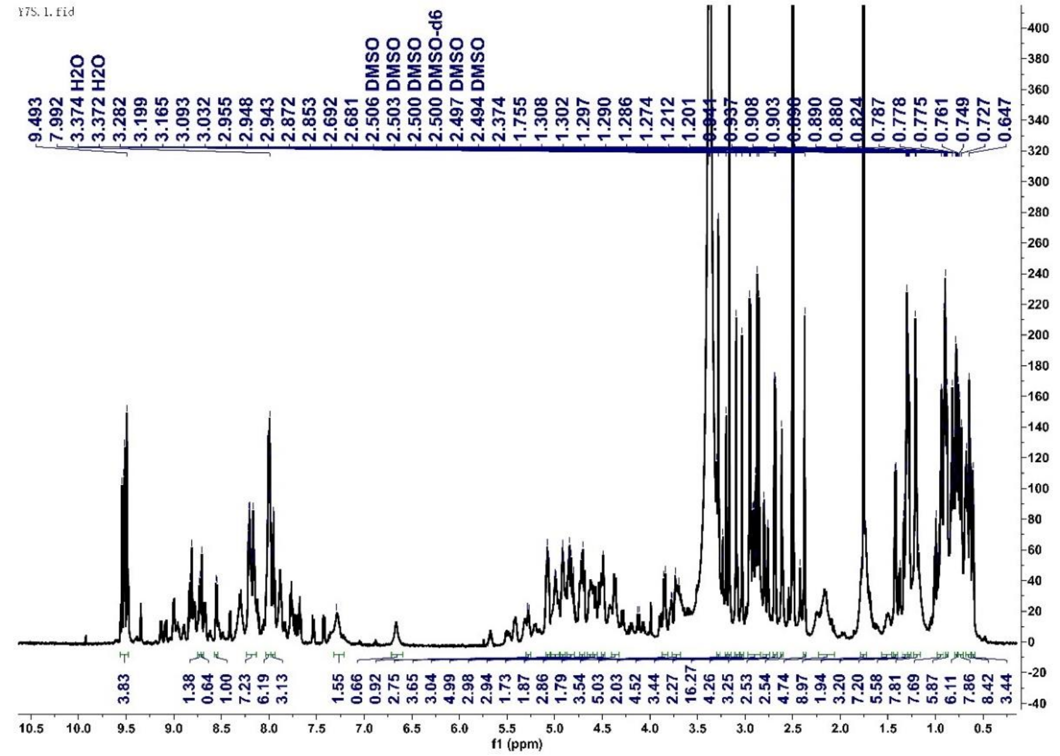

Figure S29.6 The ^1H NMR (600 MHz) spectrum of synthetic-**2** in DMSO-d_6.

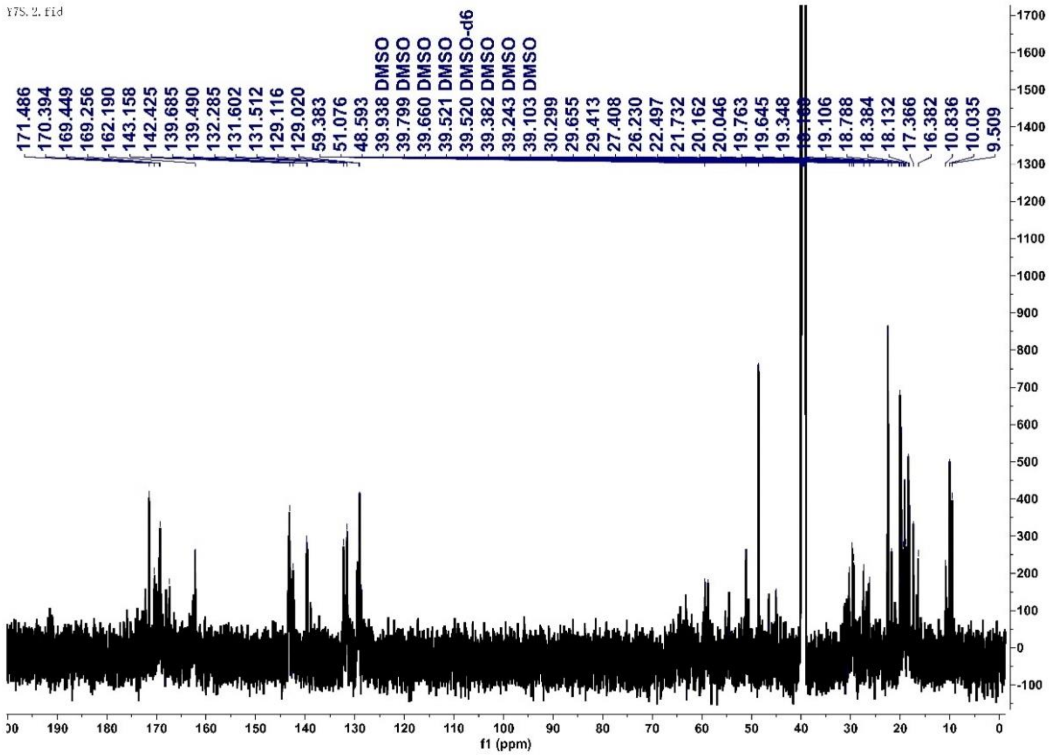

Figure S30. The ^{13}C NMR (150 MHz) spectrum of synthetic-**2** in DMSO-d_6.

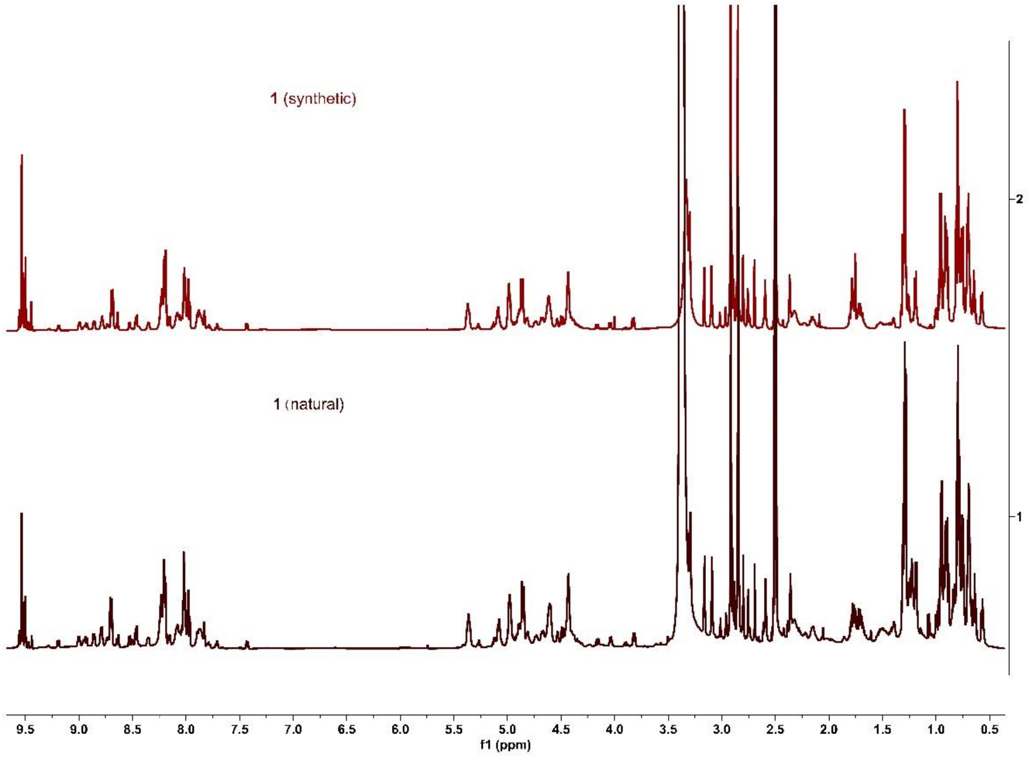

Figure S31. Comparison of ^{1}H NMR spectra (600 MHz) of natural and synthetic of **1**.

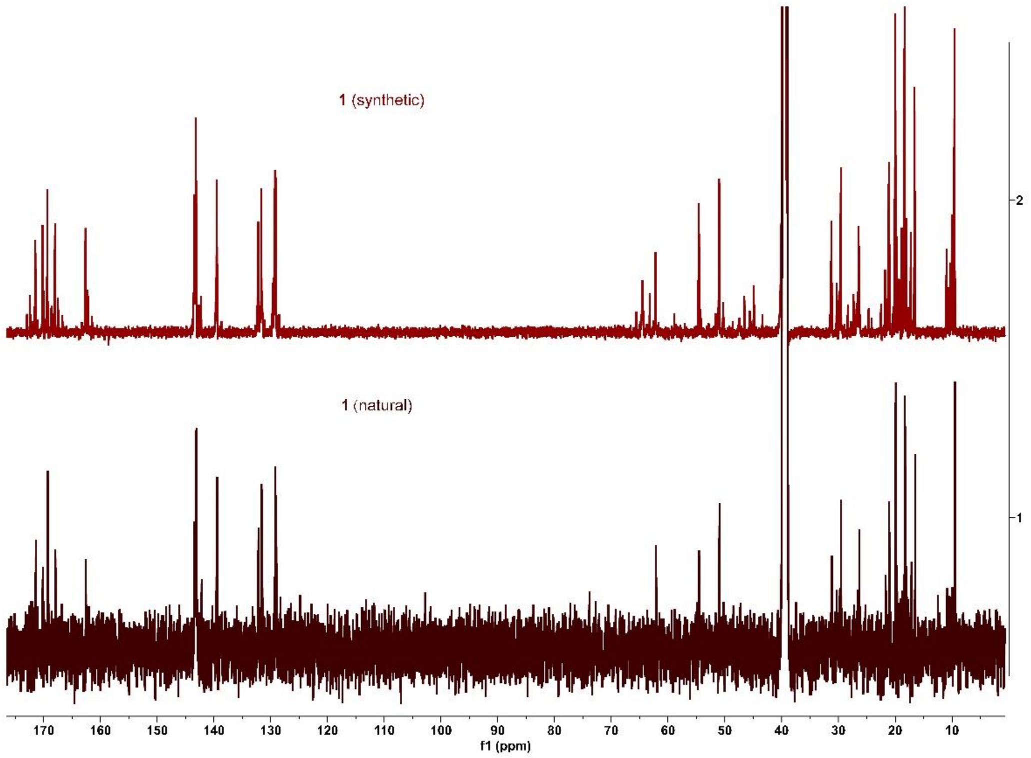

Figure S32. Comparison of ^{13}C NMR spectra (150 MHz) of natural and synthetic of **1**.

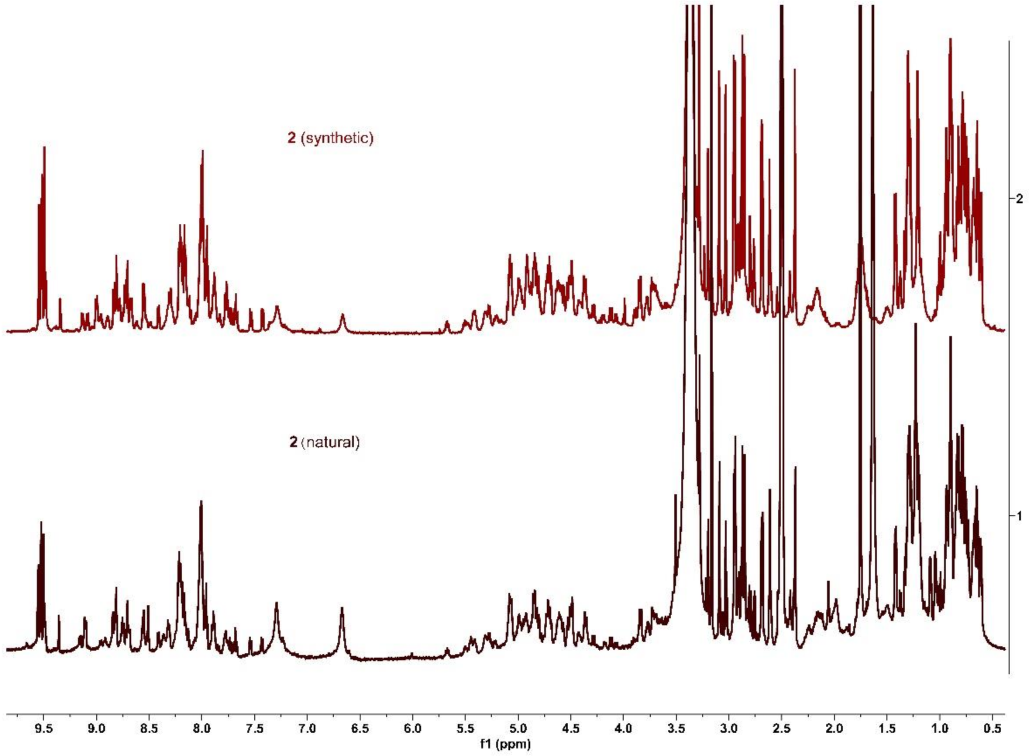

Figure S33. Comparison of ^{1}H NMR spectra (600 MHz) of natural and synthetic of **2**.

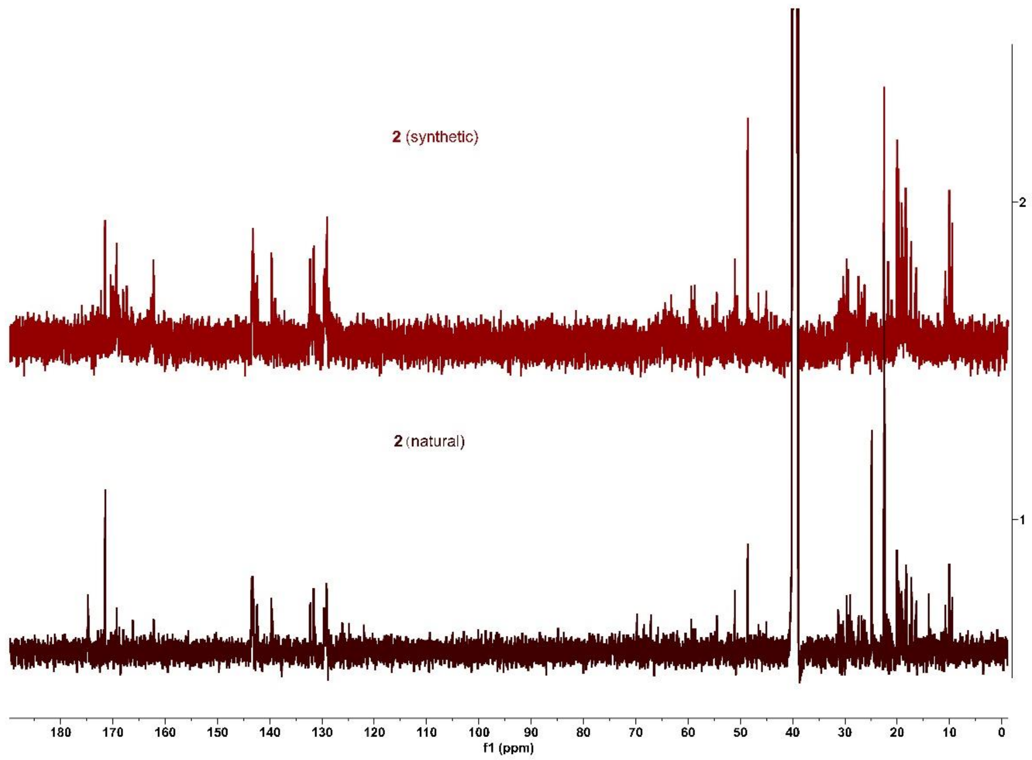

Figure S34. Comparison of ^{13}C NMR spectra (125 MHz) of natural and synthetic of **2**.

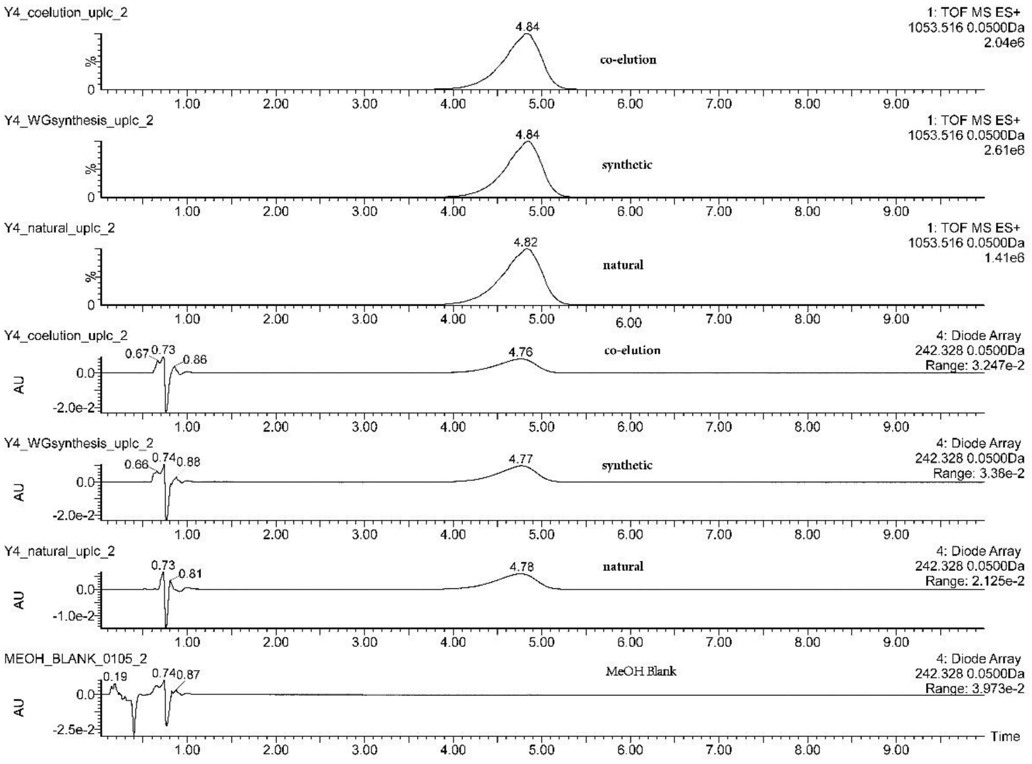

Figure S35. The UPLC-UV-MS chromatograms of **1** (natural, synthetic, and co-elution) in the condition of ACN/water with 0.1% formic acid.

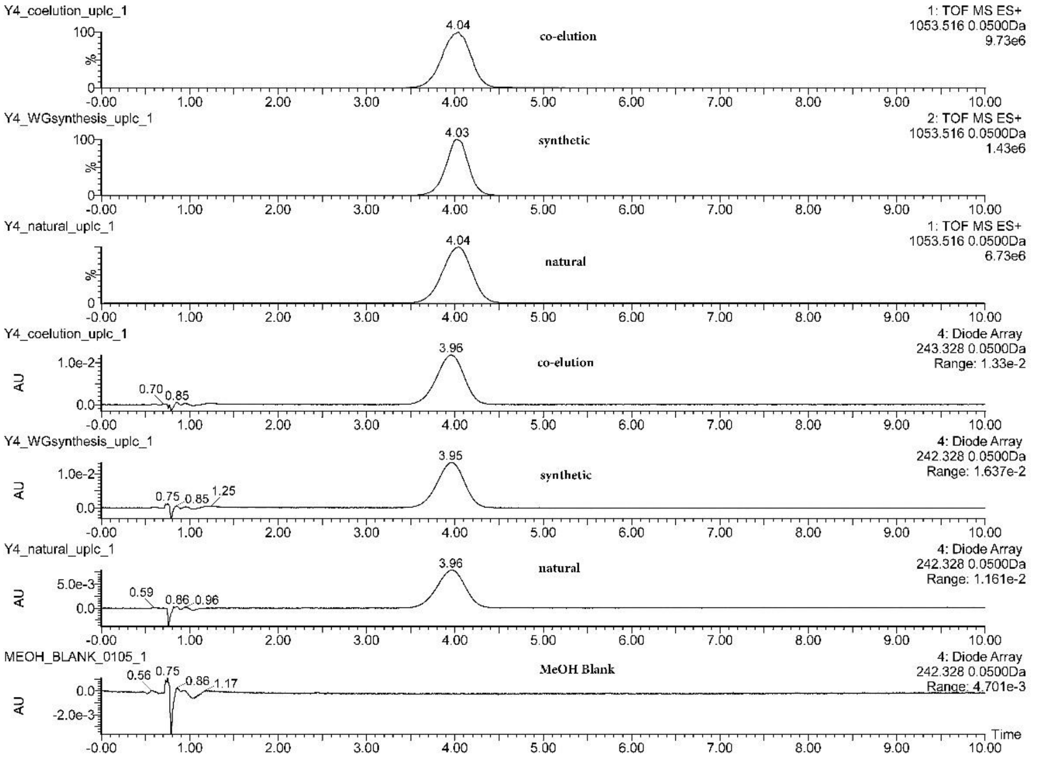

Figure S36. The UPLC-UV-MS chromatograms of **1** (natural, synthetic, and co-elution) in the condition of MeOH/water with 0.1% formic acid.

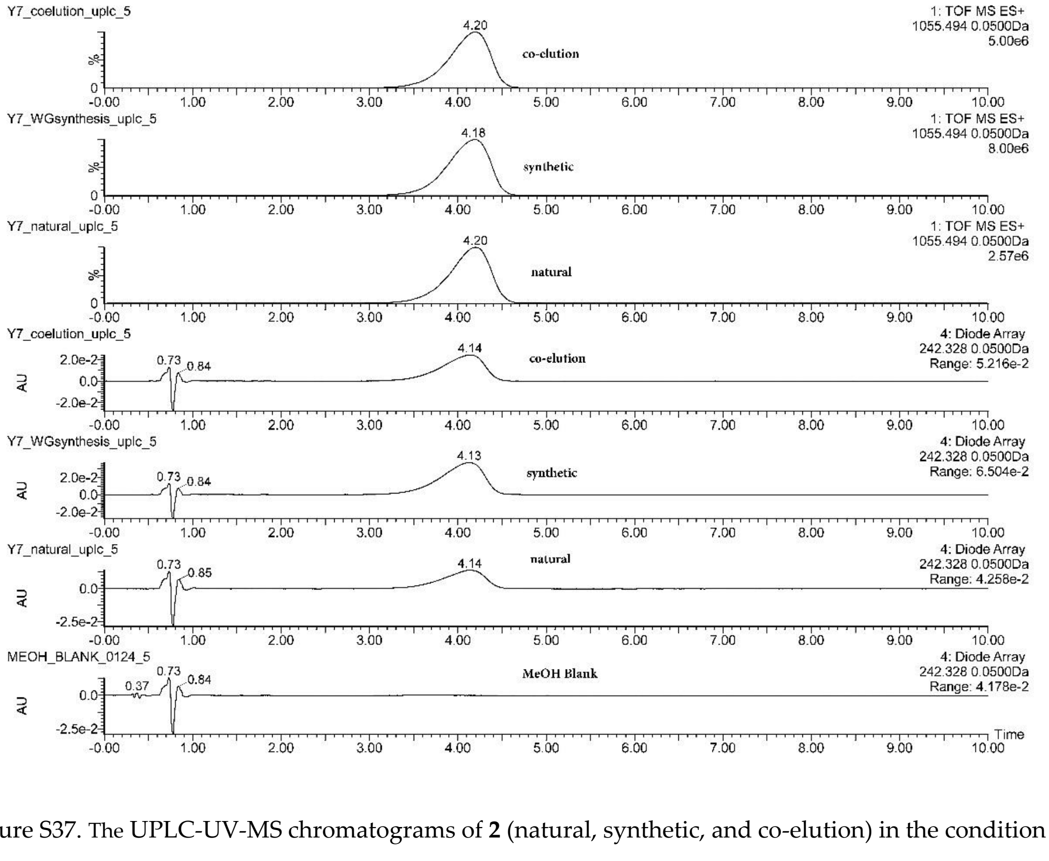

Figure S37. The UPLC-UV-MS chromatograms of **2** (natural, synthetic, and co-elution) in the condition of

ACN/ water with 0.1% formic acid.

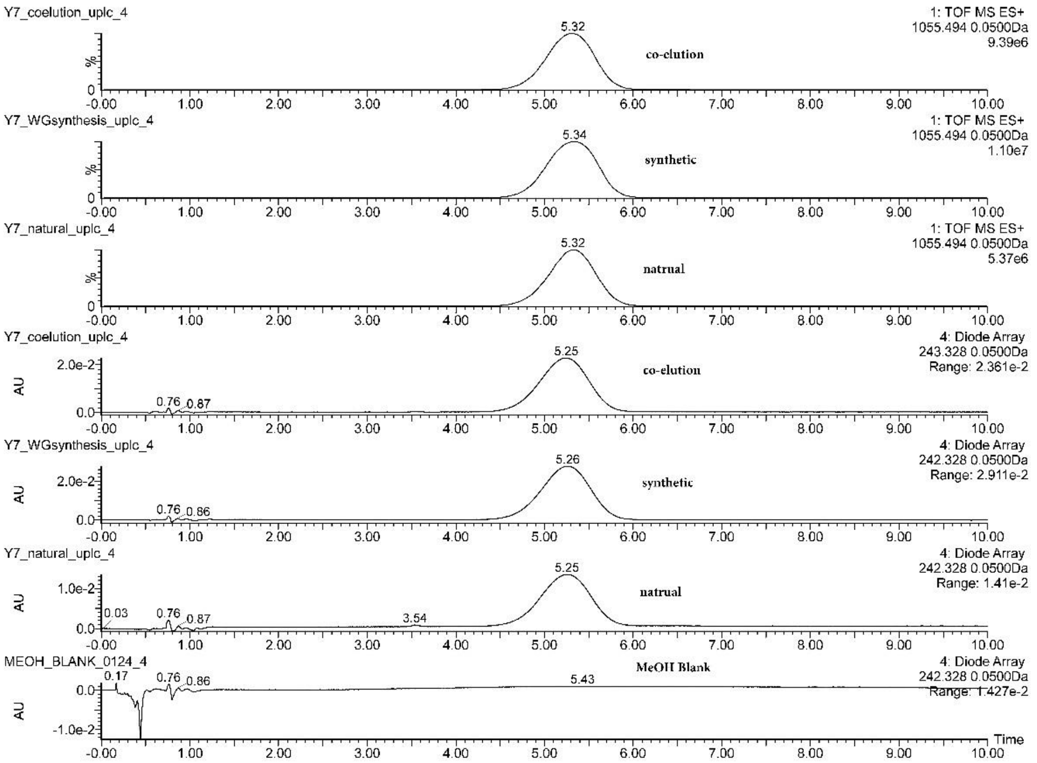

Figure S38. The UPLC-UV-MS chromatograms of **2** (natural, synthetic, and co-elution) in the condition of MeOH/ water with 0.1% formic acid.

Table S1. The physicochemical properties of natural-**1**, synthetic-**1**, natural-**2**, and synthetic-**2**.

	natural-**1**	synthetic-**1**	natural-**2**	synthetic-**2**
Properties	white amorphous powder	white amorphous powder	white amorphous powder	white amorphous powder
HRESIMS	1053.5165 $[M+H]^+$ (calcd for $C_{52}H_{69}N_{12}O_{12}$, 1053.5158)	1053.5139 $[M+H]^+$ (calcd for $C_{52}H_{69}N_{12}O_{12}$, 1053.5158)	1055.4930 $[M+H]^+$ (calcd for $C_{51}H_{67}N_{12}O_{13}$, 1055.4951)	1055.4944 $[M+H]^+$ (calcd for $C_{51}H_{67}N_{12}O_{13}$, 1055.4951)
Molecular formula	$C_{52}H_{68}N_{12}O_{12}$	$C_{52}H_{68}N_{12}O_{12}$	$C_{51}H_{66}N_{12}O_{13}$	$C_{51}H_{66}N_{12}O_{13}$
IR cm-1	3375, 3303, 2968, 2936, 2877, 1742, 1644, 1517, 1492, 1459, 1408, 1180, 1126, 1014	3378, 3312, 2968, 2937, 2877, 1743, 1644, 1517, 1492, 1465, 1408, 1180, 1128, 1014	3351, 2918, 2850, 1740, 1637, 1578, 1538, 1492, 1465, 1409, 1260, 1097, 1025	3304, 2938, 2872, 1741, 1640, 1536, 1492, 1462, 1412, 1261, 1109, 1052
UV(MeOH) λmax (log ε)	242 (4.92), 317 (4.21)	242 (4.82), 324 (4.10)	241 (4.59), 323 (3.91)	242 (4.54), 317 (3.83)
$[\alpha]_D^{25}$	-110.0° (c 0.08, ACN)	-110.0° (c 0.08, ACN)	-152.0° (c 0.05, ACN)	-155.0° (c 0.05, ACN)

Table S2. The antibacterial activities of synthetic-**1** and synthetic-**2** (MIC, μg/mL).

Test organisms	Strain No.	R/S[b]	synthetic-**1**	synthetic-**2**	Ec[c]	Lev[c]
Staphylococcus epidermidis	ATCC 12228	MSSE	>64	>32	0.25	0.25
	18-1[a]	MRSE	>64	>32	0.5	4
	17-13[a]	MSSE	>64	>32	0.12	0.25
Staphylococcus aureus	ATCC 29213	MSSA	>64	>32	0.12	0.5
	ATCC 33591	MRSA	>64	>32	0.25	0.25
	15[a]	MSSA	>64	>32	0.25	0.25
	18-2[a]	MRSA	>64	>32	0.25	>32
	18-3[a]	MSSA	>64	>32	0.12	1
	ATCC 43300	MRSA	>64	>32	0.12	0.5
	ATCC 700698	MRSA,VISA	>64	>32	0.12	16
Enterococcus faecalis	ATCC 29212	VSE	>64	>32	0.12	1
	ATCC 51299	VRE	>64	>32	0.12	1
	ATCC 51575	VRE	>64	>32	0.12	2
	18-6[a]	VSE	>64	>32	0.25	2
Enterococcus faecium	ATCC 700221	VRE	>64	>32	0.12	>32
	18-4[a]	VSE	>64	>32	0.25	4
	15-6[a]	VRE	>64	>32	0.25	>32
Escherichia coli	ATCC 25922	ESBLs(-)	>64	>32	8	0.06
	ATCC 35218	ESBLs(+)	>64	>32	1	0.06
	1515[a]	ESBLs(-)	>64	>32	2	0.06
	18-1[a]	ESBLs(-)	>64	>32	8	2
	18-4[a]	ESBLs(+)	>64	>32	16	16
Klebsiella pneumoniae	ATCC 700603	ESBLs(+)	>64	>32	32	1
	ATCC BAA-2146	NDM-1(+)	>64	>32	16	>32
	7[a]	ESBLs(-)	>64	>32	>32	0.12
	18-2[a]	ESBLs(-)	>64	>32	>32	0.25
	18-8[a]	ESBLs(+)	>64	>32	>32	1
Pseudomonas aeruginosa	ATCC 27853		>64	>32	>32	2
	PAO1		>64	>32	>32	1
Acinetobacter baumannii	ATCC 19606		>64	>32	4	0.5
	16-33[a]	CRAB	>64	>32	8	4
Enterobacter cloacae	ATCC 43560		>64	>32	4	≤0.03
Enterobacter aerogenes	ATCC 13048		>64	>32	16	0.12
Serratia marcescens	ATCC 21074		>64	>32	>32	0.25
Proteus mirabilis	ATCC 49565		>64	>32	>32	0.12
Stenotrophomonas maltophilia	ATCC 13636		>64	>32	4	2
Shigella flexneri	ATCC 12022		>64	>32	8	≤0.03

[a]clinical isolates; [b]drug-resistance; MSSE, methicillin-susceptible *S. epidermidis*; MRSE, methicillin-resistant *S. epidermidis*; MSSA, methicillin-susceptible *S. aureus*; MRSA, methicillin-resistant *S. aureus*; VISA, Vancomycin-intermediate *S. aureus*; VSE, Vancomycin-sensitive *Enterococcus*; VRE, Vancomycin-resistant *Enterococcus*; ESBLs, extended-spectrum beta-lactamases; NDM-1, New Delhi Metallo-beta-lactamase-1; CRAB, Carbapenem-resistant *A. baumannii*; [c]positive control, Ec, echinomycin, Lev, Levofloxacin.

Table S3 The cytotoxic activity of synthetic-**1** and synthetic-**2** against H460 lung cancer cells

	synthetic-**1**	synthetic-**2**	**echinomycin**
IC_{50} (nM)	>1000	>1000	1.5 ± 0.2

IC_{50}, 50% inhibitory concentration (mean ± SD, n=3). They were determined by MTT assay following 72 h of drug treatment.
